# Regeneration of Insulin-Producing β Cells, Reduction in Inflammation and Oxidation Stress, and Improvement in Lipid Profile in a Type 1 Diabetes Rat Model by Intraperitoneal Injection of the Growth Factors-Rich Catfish Skin-Derived Fraction-B: An Introductory Report

**DOI:** 10.3390/biom15070929

**Published:** 2025-06-25

**Authors:** Jassim M. Al-Hassan, Waleed M. Renno, Sosamma Oommen, Divya Nair, Bincy Maniyalil Paul, Bincy Mathew, Jijin Kumar, Afna Ummerkutty, Cecil Pace-Asciak

**Affiliations:** 1 Health Sciences Research Centre, Khaldiya Campus, Abdullah Al-Salem University, P.O. Box 2249, Safat 13023, Kuwait City, Kuwait; sosamma.oommen@aasu.edu.kw (S.O.); bincy.mathew@aasu.edu.kw (B.M.); afna.ummerkutty@aasu.edu.kw (A.U.); 2 Biological Sciences Department, Faculty of Science, Shedadiya Campus, Kuwait University, P.O. Box 5969, Safat 13060, Kuwait; divyajs2002@yahoo.co.in (D.N.); bincymaniyalil@gmail.com (B.M.P.); 3 Department of Anatomy, Faculty of Medicine, Kuwait University, P.O. Box 24923, Safat 13110, Kuwait City, Kuwait; waleed.renno@ku.edu.kw (W.M.R.); jijin.kumar@imperial.ac.uk (J.K.); 4 Translational Medicine, Research Institute, The Hospital for Sick Children, Toronto, ON M5G OA4, Canada; cecil.pace-asciak@sickkids.ca; 5 Department of Pharmacology, Faculty of Medicine, University of Toronto, Toronto, ON M5S 1A8, Canada

**Keywords:** diabetes, catfish, Fraction-B, β-cells, stem cells, lipids, proteins, growth factors, regeneration

## Abstract

Type 1 diabetes (T1D) results from the autoimmune destruction of insulin-producing β-cells. The regeneration of durable insulin-producing β-cells remains a critical challenge. This study investigated the regenerative potential of Fraction-B (FB), a catfish skin-derived preparation rich in growth factors, in a T1D rat model to regenerate active β-cells. Sprague Dawley rats with T1D caused by streptozotocin injection received daily intraperitoneal injections of FB for 8 weeks. FB treatment significantly reduced blood glucose to a level close to that of normal control animals, increased serum insulin and C-peptide, and restored pancreatic insulin content. Histopathological and immunohistochemical analyses confirmed the regeneration of insulin-producing β-cells in pancreatic islets. FB treatment also improved diabetes-related health issues through a reduction in inflammation and oxidative stress, and an improvement in lipid profiles without toxicity or side effects. The regenerated β-cells remained functional for 48 weeks without the use of immunosuppressants, until the animals were sacrificed. These findings suggest FB treatment to be a promising procedure for translational research into T1D treatment.

## 1. Introduction

Type 1 diabetes (T1D) is an autoimmune disease characterized by the destruction of pancreatic β cells, leading to insulin deficiency and chronic hyperglycemia. This condition causes severe complications, including cardiovascular disease, neuropathy, retinopathy, kidney failure, and diabetic foot ulcer, significantly impacting quality of life [1]. The destruction of β cells in children and in adults leads to T1D [2]. It is well established that not all β cells in T1D are destroyed. The residual β-cell numbers are small and are reported to be approximately 5–10% of the original number of cells. Whether the number of the residual β cells is too small, scattered, or they are not producing enough insulin, this results in T1D [3]. Current T1D management relies on exogenous insulin, which controls blood glucose but does not restore β-cell function or prevent long-term complications. It is theoretically possible for regeneration to occur through the stimulation of β-cell replication or the conversion of other pancreatic cells (α-, δ-, or ε-cells) into β cells. As the adult pancreas has limited regenerative potential, the research has focused on β-cell regeneration or transplantation to cure diabetes. Regenerating durable, insulin-producing β cells is a critical goal for curing T1D yet achieving this is still challenging [4]. The existing approaches, such as stem cell therapies, show promise, but face hurdles in scalability, safety, immune rejection and with ramifications, while whole pancreas or pancreatic islet transplantation is constrained by donor scarcity, high costs, a high morbidity rate, and the need for lifelong immunosuppression [5,6,7]. These limitations highlight the urgent need for innovative, cost-effective strategies to regenerate functional β cells without immunosuppressive requirements.

Fraction-B (FB), prepared from the skin of the catfish *Arius bilineatus, Val*. is a potent wound healing material. It offers a novel approach to β-cell regeneration, as it is rich in bioactive lipids and proteins. The protein fraction is involved in wound healing [8]. The lipid fraction of FB contains components that exhibit anti-inflammatory, antioxidant, anti-cancer, and regenerative properties, and cause NETosis, such as cholesterol oxidized derivatives and furan fatty acids [9,10,11,12]. Previous studies have demonstrated FB’s efficacy in promoting wound healing in non-healing diabetic foot ulcers, regenerating crushed sciatic nerves in rats, and the treatment of pancreatic cancer in mice without toxicity or side effects [8,13,14,15,16].

This study investigates whether FB can regenerate insulin-producing β-cells in a streptozotocin (STZ)-induced T1D rat model. We hypothesize that FB’s growth factor-rich composition, combined with its anti-inflammatory and antioxidant properties, will heal the STZ-injured pancreas, promote β-cell regeneration, and restore glycemic control. Type 1 diabetic Sprague Dawley (SD) rats received daily intraperitoneal (IP) FB injections for 8 weeks. This introductory report evaluates FB’s effects on pancreatic recovery, active insulin-producing β-cell regeneration, and associated health benefits, including a reduction in inflammation and oxidative stress and the improvement of lipid profiles in the treated diabetic animals. It also analyzes FB for growth factors and active proteins that might contribute to the recovery of the diabetic pancreas and regeneration of the insulin-producing β-cells. To achieve this, we employed biochemical, histopathological, and immunohistochemical methods. This study is driven by FB’s regenerative effects, its ability to stimulate tissue repair, and its ability to modulate inflammation and oxidation stress, suggesting its potential to restore pancreatic function in T1D animals, an application not previously explored.

## 2. Materials and Methods

### 2.1. Preparation of Fraction B from Catfish Skin

Catfish epidermal secretions were collected, and catfish skin preparation (CSP) was performed as described previously [8,13,17]. FB was prepared from CSP utilizing the procedures described in our two published US patents [18,19]. FB was prepared in PBS for injection into the diabetic animals. FB was administered in mls of PBS containing FB calculated to have 3 mg of protein of FB/kg animal body weight. For the determination of the concentration of proteins in FB, the FB sample was diluted with PBS. A total of 0.1 mL of the diluted sample was mixed well with 5 mL of Coomassie Brilliant Blue solution [20] and kept in tubes at room temperature for approximately 10 min. The absorbance of the sample was read at 595 nm, and its protein concentration was determined by comparing its absorbance against the absorbance of a standard curve for different bovine serum albumin concentrations.

The lipid percentage of FB was calculated using the methods described previously [14,17].

### 2.2. Experimental Animals

Male animals are commonly used in biomedical experiments because of the issue of interference from female hormones in the results. In this study, healthy male SD rats were used for all experiments. At the end of the experiments involving male animals, some of the experiments were repeated on female animals to test whether FB acts similarly in female SD rats as it did in the male animals. Three-month-old rats (150–180 g) were used throughout this study. The animals were kept under constant temperature (23 °C ± 2 °C) and humidity, with a 12 h light/dark cycle. Rats were housed individually in separate cages and were provided with food and water ad libitum throughout the experiment. Each experiment included 31 rats that were randomly divided into three groups: 7 in the normal control (NC), 12 in the diabetic control (DC), and 12 in the FB injected diabetic animals (D + FB). The animal experiments followed the procedures of the Animal Ethics Committee of Kuwait University Health Sciences Center, and this study was carried out under US guidelines (NIH Publication #85-23, revised in 1985). All efforts were made to minimize the number of animals used in this study and their suffering.

### 2.3. Establishment of Diabetes in Male Rats and Treatment with FB

Diabetes was induced in SD rats by IP injection of a single dose of 60 mg STZ/kg body weight in citrate buffer (0.01 M, pH 4.5) within 5 min of preparation [21,22]. One week after STZ injection, BG was measured, and rats with BG levels > 15 mmol/L were considered diabetic. Diabetic rats were randomly divided into two groups. One group was the DC control animals, while the second was the diabetic (D) group to be treated with FB (D + FB). The rats in the DC group were intraperitoneally (IP) injected daily with PBS (pH 7.5) for 8 weeks. The volume varied for each animal according to its weight as if it was to be injected with FB in PBS. The rats in the D + FB group were IP injected daily with FB (0.3 mg protein/100 g body weight) in PBS buffer (pH 7.5) for 8 weeks. Rats in the NC group were not diabetic (i.e., had not been treated with STZ), and were IP injected daily with the appropriate volume of PBS (pH 7.5), calculated according to the weight of each rat, as for the DC group, but without FB. All animal groups were treated at the same time of day.

The most suitable concentration of FB used in two separate published studies not related to diabetes was 3 mg FB protein/kg animal body weight [15,23]. The following FB concentrations were tested in the different studies: 1.5 mg, 3 mg, 4.5 mg, and 6 mg FB/kg animal body weight on 10–12 animals each time.

Note: The FB dose of 3 mg/kg animal body weight was adopted based on its established efficacy in prior applications. Specifically, this dose was used successfully in clinical topical treatments for non-healing diabetic foot ulcers, and in preclinical studies demonstrating sciatic nerve regeneration in rats [15,23], as well as for treating pancreatic cancer in mice [16]. The 3 mg/kg dose was selected as the optimal concentration for this study due to its consistent therapeutic effects and resource efficiency in these prior studies.

### 2.4. Evaluation of General Health Factors After FB Injection

BG levels, food and water intake, and changes in animal body weights were assessed to gauge the progress of FB treatment. BG levels were measured weekly in all three groups of animals. A drop of tail blood was collected under mild diethyl ether sedation after overnight fasting (12–14 h) and then evaluated for BG using a One-Touch Ultra-Easy Glucometer (LifeScan, CA, USA). Throughout the experiment, daily water and food intake were monitored. All rats were weighed before the start of the experiment and then weekly throughout the 8-week experimental period. To confirm the FB treatment results, the FB treatment experiment was repeated 3 times on diabetic male SD rats without changing the experimental conditions. The selection of blood and organs from test animals for all the examinations shown in Results was unbiased.

### 2.5. Animal Sacrifice and Collection of Blood and Tissue Samples

After the eight weeks of FB treatment, the rats were sacrificed under anesthesia [ketamine and xylazine (9:1) using 0.1 mL/100 g body weight] after overnight fasting. Blood was collected immediately by cardiac puncture, placed into tubes for serum preparation, and allowed to clot at room temperature. The blood was then centrifuged at 3000 rpm for 15 min. The serum was separated and stored as 0.3 mL aliquots in Eppendorf tubes at −80 °C until analysis. Moreover, the pancreas, kidneys, and liver were removed from each animal, washed well with TRIS buffer to remove blood, pressed between filter papers, and then stored at −80 °C until analysis.

### 2.6. Investigation of the Activities of the Recovered Pancreas (Production of Insulin and C-Peptide)

The selection of the organs from the animals within the three groups of test animals for the investigation of the activities of the recovered pancreas was unbiased. For the determination of the factors underlying the notable reduction in BG in the FB-treated diabetic animals, insulin was measured in both the serum and the homogenized pancreatic tissue of the three groups of experimental animals, while the C-peptide was measured in the sera of the D + FB animals and compared with those in the NC and DC animals.

#### 2.6.1. Analysis of Insulin in the Serum

The serum insulin concentration was determined in each of the three groups of sacrificed animals using a rat-specific ELISA kit (SPI Bio, Bertin Pharma, Montigny-le-Bretonneux, France) following the instructions provided with the kit without modification.

#### 2.6.2. Analysis of Fructosamine in the Serum

As fructosamine concentration in serum reflects the degree of glycation of serum proteins as the hemoglobin A1c (HbA1c) level does, we measured the fructosamine level in the serum of each of the three sacrificed animal groups after eight weeks of treatment, following the method of Chung et al. (1988) [24].

#### 2.6.3. Analysis of Insulin in the Pancreatic Tissue

To quantify the total pancreatic insulin content without cell loss, the pancreatic tissue of each of the three sacrificed groups of animals was homogenized and treated as follows: PBS (9 mL) was added to 1 gm pancreatic tissue. Protease inhibitor (e.g., 1 mM PMSF) was then added as provided with the kit. The sample was then homogenized by twice freezing and thawing processes. The homogenate was then centrifuged for 5 min at 5000 rpm× *g*. The supernatant was then immediately collected and measured for insulin concentration, using a rat-specific ELISA kit (Wuhan Fine Biotech Co., Wuhan, China) following the instructions provided with the kit without modification. The procedure for measuring the total concentration of insulin in the pancreas was also used by others [25].

#### 2.6.4. Determination of C-Peptide in the Serum

The serum C-peptide level indicates the activity of the pancreas. C-peptide levels in the blood serum of the three groups of animals were determined using a rat C-peptide ELISA kit (Catalog No. E-El-R0032 by Elabscience; Houston, TX, USA) following the instructions provided with the kit without modification.

### 2.7. Histopathology of the Pancreas and Immunohistochemical Staining for Insulin in the Islets

#### 2.7.1. Histopathology of the Pancreas

To establish that FB treatment caused the recovery of the islets and that the new islets contained insulin-producing β cells, pancreatic tissues from the three groups of sacrificed animals were processed for histopathological and immunohistochemical studies. Histopathological (5-micron thickness) and immunohistochemical (4-micron thickness) sections of pancreatic tissues from each of the three experimental groups were cut for examination using a Leica manual rotary microtome (Rm2235). The histological sections were stained with Mayer’s hematoxylin and eosin (Sigma-Aldrich, London, UK), as previously described [26,27,28], and were then viewed under a light microscope.

#### 2.7.2. Immunohistochemical Staining for Insulin

To establish that the newly developed structures seen in the light microscopic sections of the FB-treated pancreases of the FB-treated rats were recovered islets of Langerhans and that they contained insulin-producing β cells, pancreatic tissues from the 3 groups of animals (randomly selected 3 animals/group) were processed for immunohistochemistry using Al-Adwani’s procedure [27] as follows: The pancreatic tissues were sliced into 3 sections, each measuring 1 mm thick, and random sections of 4 μm thickness were cut (10 sections per rat). These sections were subsequently mounted on Poly-L-lysine coated glass slides and allowed to dry overnight. The sections were immunostained using an insulin primary antibody (2D11-H5; sc8033; Santa Cruz Biotechnology, San Diego, CA, USA) and incubated overnight. Following this, the sections were treated with a biotinylated secondary antibody conjugated to horseradish peroxidase (goat anti-mouse IgG-HRP: sc-2031, Santa Cruz Biotechnology, San Diego, CA, USA). After applying a chromogen solution containing 3-diaminobenzidine (DAB) (DAB kit, SK-4100, Vector Labs, Burlingame, CA, USA), the sections were counterstained with hematoxylin (Cat No. H9627-100G; Sigma, Saint Louis, MI, USA). Finally, the slides were examined using an Olympus microscope (DP-72; Olympus, Tokyo, Japan).

### 2.8. Improvement of Health Issues Related to Diabetes

To evaluate FB’s effect on diabetes-caused oxidative stress, inflammation, and alteration in the lipid profiles, each of these three health issues was investigated after FB treatment.

#### 2.8.1. Effect of FB Treatment on Oxidation/Antioxidation in the Kidneys and Liver

Oxidative stress manifests in the kidneys and liver. To establish the oxidative/antioxidative activities of FB in the FB-treated diabetic animals, experiments were performed on homogenized kidneys and livers from the 3 experimental animal groups as follows.

##### Concentration of MDA

MDA levels reflect lipid peroxidation. The MDA concentration was measured in both the homogenized kidneys and livers of each of the three groups of animals separately using the method of Ohkawa et al. [29].

##### Concentration of Antioxidants

The concentrations of antioxidants in the homogenized kidneys and livers from each of the three groups of animals were determined using the method of Drobiova [30].

##### Concentration of Catalase

Catalase is a key enzyme that uses hydrogen peroxide, a nonradical ROS, as its substrate. The method of Aebi [31] was used to measure the catalase concentration in the kidneys and livers of each of the three groups of experimental animals.

#### 2.8.2. Effect of FB Treatment on Inflammatory Cytokine

FB contains F-acids, S5, and IL19. These agents are anti-inflammatory, and their presence was expected to exert anti-inflammatory effects on the D + FB animals. To establish whether FB had an influence on the inflammation in the FB-treated diabetic animals, pro- and anti-inflammatory cytokines in the three groups of animals were analyzed. Pro- and anti-inflammatory cytokine levels in the blood sera of the three groups of animals were measured using a Rat Specific Multianalyte ELISA Qiagen analysis kit (Qiagen Sciences, Beverly, MA 20874, USA). Analysis for the levels of the proinflammatory cytokines IL1α, IL1β, IL4, IL6, IL12, IL13, TNFα, GMC-SF, and RANTES, and the anti-inflammatory cytokines IL2 and IFNγ was performed using this kit. The proinflammatory cytokine IL6 and the anti-inflammatory cytokine IL10 could not be detected with the same kit, so blood sera from each of the three groups were analyzed using an IL6 Invitrogen kit (cat # BMS625) and an IL10 Invitrogen kit (cat. # BMS629) (both from Thermo Fisher Scientific, Bender MedSystems GmbH, Campus Vienna Biocenter 2, Vienna, Austria). The instructions provided by the manufacturer for each kit were followed without modification.

#### 2.8.3. Effect of FB Treatment on Lipid Profile

To determine the effects of FB on the altered lipid profiles [32] in the diabetic animals, the concentrations of serum lipids (cholesterol, HDL, LDL, and triglycerides) in the three groups of animals were determined.

Cholesterol, HDL, and triglyceride levels were measured using kits from Randox Laboratories Limited, Crumlin, UK, without modification (Ref CH201, lot 452752; Ref CH2655, lot 431831; and Ref TR212, lot 483735, respectively). The LDL concentration was calculated using the Friedewald equation as follows:

Total cholesterol = LDL cholesterol + HDL cholesterol + triglycerides/5

### 2.9. Evaluation of Safety of FB Treatment

To evaluate the safety of FB treatment, normal non-diabetic (NC) SD male rats were subjected to daily IP administration of 3 mg FB/kg body weight for 16 weeks instead of the standard 8-week treatment. Their body weight and BG were recorded. They were then sacrificed under anesthesia and their organs were examined for gross pathological changes.

### 2.10. Investigation of the Durability of the Regenerated β Cells After FB Treatment

The durability of the regenerated β-cells in insulin production is an important factor when judging the recovery of the pancreas and for future human application of the FB-treatment procedure to diabetic patients. For this reason, the durability of the regenerated β-cells in insulin production after FB treatment was investigated. Three groups of SD rats were included: the NC group, DC group, and D + FB group. Each group contained 31 animals (NC = 7; DC = 12; D + FB = 12). The FB-treated diabetic animals received FB treatment for eight weeks, as previously described in the other experiments. Their BG and weights were compared to those of the NC and DC groups, which were treated with buffered saline as previously described in the other experiments. At the end of week eight, the NC and DC animals were sacrificed under anesthesia, and their sera were evaluated for insulin and C-peptide concentrations. For the D + FB animals, FB treatment was halted at the end of week eight, after which the animals were kept under observation for 40 weeks. During the 40-week period, BG and weight measurements were continued weekly. At the end of 48 weeks (8 + 40), the D + FB animals were sacrificed under anesthesia, and their sera were evaluated for insulin and C-peptide concentrations. No immunosuppressants were administered to the D + FB animals.

### 2.11. Analysis of FB for Growth Factors and Biologically Active Proteins That Can Play Roles in the Recovery of the Diabetic Pancreas and Regeneration of β-Cells

FB was not analyzed for growth factors in our previous studies. In this study, FB was analyzed for several growth factors that play roles in wound healing, the improvement of diabetes conditions, and regeneration. In this study, specific ELISA kits for rats were used for the determination of the presence of homologous factors that act in a similar fashion to that of each protein of interest in the fish material, following the manufacturer’s instructions for each kit without modification. In anticipation of future applications of FB for human treatment, specific ELISA kits were used for the search for homologous and equivalent human growth factors in FB that might have similar actions in humans as those in rats. FB was analyzed for the presence of 81 proteins of interest. As no rat or human growth factors exist in the fish materials (FBs), the selected proteins were called rat or human growth factors for simplicity of identification. Table 1 lists each growth factor and active protein of interest accompanied by the ELISA kit used for its identification and the reference indicating its known biological function.

### 2.12. FB Treatment of Diabetic Female SD Rats Is Given in Supplementary Material

This study used male SD rats as an animal model. To test the effects of FB on diabetic female SD rats, the animals were injected with STZ as detailed for the males in Section 2.3 above [21,22]. Several of the experiments that were performed on diabetic male SD rats were repeated on diabetic female SD rats using procedures similar to those applied to the male animals.

### 2.13. Unified Graph Legend and Significance Symbols

In all figures shown in the text and those shown in the Supplementary Materials, the symbols of significance are as follows: a = significantly different from NC; b = significantly different from DC; and c = not significantly different from NC; a, b indicates that a is significantly different from NC, and b is significantly different from DC; b, c indicates that b is significantly different from DC, while c is not significantly different from NC. 

Graph legend symbols for line graphs are as follows: 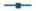 NC = normal control; 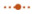 DC = diabetic control; and 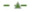 D + FB = fraction B-treated diabetic rats. Graph legend symbols for bar graphs are as follows: 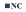 = normal control; 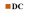 = diabetic control; and 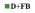 = fraction B-treated diabetic rats.

### 2.14. Statistical Analysis

The data are presented in line and bar graphs as the mean ± SEM of the absolute values. Statistical differences among the three groups were calculated using one-way ANOVA (SPSS, V 22, IBM, New York, NY, USA) with the LSD post hoc test, with *p* < 0.05 indicating statistical significance. Differences among the three groups at each corresponding stage of the experiment are also presented as percentages in the Results Section.

## 3. Results

### 3.1. Benefits of FB Treatment to Diabetic Animals

#### 3.1.1. FB Treatment Caused a Reduction in BG Levels in the Diabetic Animals

**Figure 1:**
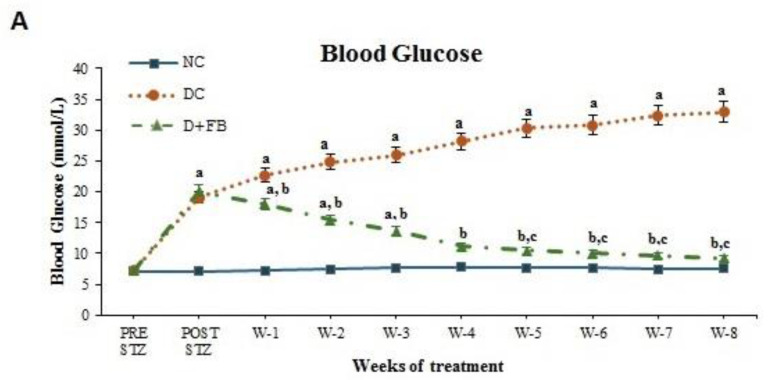

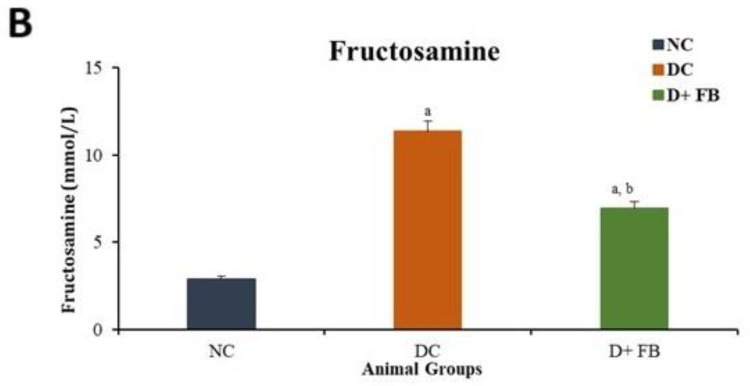
(**A**,**B**) FB treatment caused a reduction in BG and fructosamine levels in the serum of the treated diabetic animals. (**A**) Line graph shows the significant reduction in the BG level. (**B**) Bar graph shows fructosamine level in the serum of the D + FB-treated male rats. Both results shown in graphs (**A**) and (**B**) are significant (*p* < 0.05) compared to those in the DC and NC rats at the end of 8 weeks of FB treatment.

For the determination of the effect of FB treatment on BG levels in the treated diabetic animals, the average BG level of the three animal groups [normal control (NC), diabetic control (DC), and diabetic rats to be treated with FB (D + FB)] was measured before STZ injection in the DC and D + FB groups and was found to be 7.2 mmol/L at week 0. The NC animals were injected with PBS at a volume equal to that of STZ (injected in the other two groups), while the DC and D + FB groups were injected with a calculated volume of STZ as described in the Materials and Methods Section, Section 2.3. The average BG concentration after seven days of STZ injection in the DC animals was 18.90 mmol/L, and that in the D + FB animals was 20.90 mmol/L. The animals in the D + FB group were IP injected daily with FB (3 mg FB/kg animal body weight), while the animals in the NC and DC groups were injected with an equivalent volume of PBS. BG levels were measured in the three groups of animals once a week throughout the eight-week experiment. The NC group showed the same average BG level (7.2 mmol/L) throughout the experiment. The DC group showed a weekly increase in BG levels, ultimately reaching 33.00 mmol/L (an increase of 334% above that in the NC animals) at the end of week eight. In contrast, the BG levels in the D + FB group were close to those in the DC group (20.90 mmol/L) at week 0 and then showed a gradual decrease. By week eight, the average BG level in the D + FB animals was close to that in the NC animals (9.2 mmol/L) and was significantly (*p* < 0.05) reduced by 72% compared to that in DC animals (Figure 1A). FB treatment resulted in a remarkable reduction in BG concentration in the D + FB group of animals, as noted by the end of the first week, and then continued until the end of week 8. These results show the effectiveness of FB treatment in reducing BG in diabetic animals and are indicative of the recovery of the pancreas. The reduction in BG levels in these animals over such a short period indicates the efficacy of FB treatment in inducing the recovery of the diabetic pancreas.

#### 3.1.2. FB Treatment Caused a Reduction in Serum Fructosamine in the Diabetic Animals

The fructosamine levels in the serum of each of the three sacrificed animal groups were measured after eight weeks of FB treatment, following the methods of Chung et al. (1988) [24]. The average serum fructosamine level in the NC animals was 2.90 ± 0.712 mmol/L. The average fructosamine concentration in the DC animals significantly increased to 11.36 ± 0.698 mmol/L (291% increase above that in the NC animals). However, the FB-treated diabetic animals had a fructosamine level of 6.25 ± 0.457 mmol/L, an 115% increase compared with that of the NC group and a significant 55% reduction compared with that of the DC group, indicating the recovery of the diabetic pancreas. The reduction in fructosamine concentration in the D + FB group of animals is indicative of the reactivation of the diabetic pancreas through the action of FB. For all the obtained values, the level of significance was *p* < 0.05 (Figure 1B).

#### 3.1.3. FB Treatment Improved the Body Weight of the Diabetic Animals

**Figure 2:**
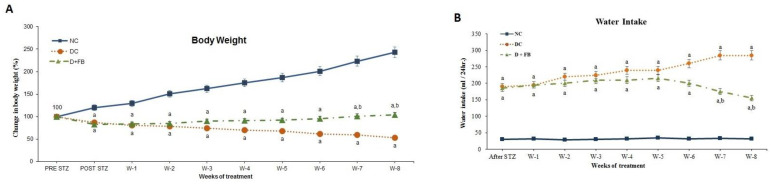

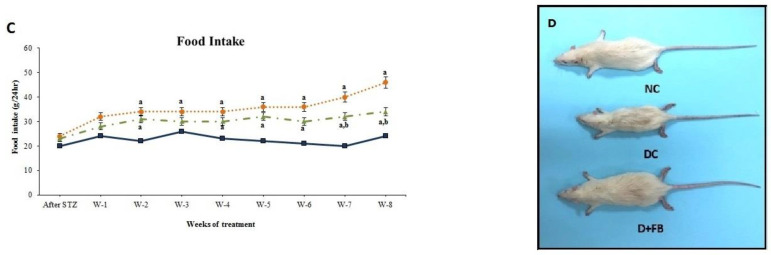
(**A**–**D**) FB treatment improved the body weight, reduced the water intake, reduced the food intake, and improved the physical appearance of the treated diabetic animals. (**A**) A line graph showing the significant improvement in body weight. (**B**) A line graph showing the significant reduction in water intake. (**C**) A line graph showing the significant reduction in the food intake of the D + FB-treated male rats. All are significant (*p* < 0.05) compared to those in the DC and NC rats at the end of 8 weeks of FB treatment. (**D**) A photograph showing the improvement in physical appearance at the end of 8 weeks of FB treatment. The FB-treated animal looks healthy, like the NC animal, at the end of 8 weeks of FB treatment.

The weights of the animals in the three groups were measured weekly during the 8-week treatment period. The DC animals showed a gradual decrease in body weight compared to that of the NC animals, while the FB-treated animals showed a significant increase in body weight compared to that of the DC group, indicating a beneficial effect of FB treatment and the alleviation of diabetes in the treated animals. For all the obtained values, the level of significance was *p* < 0.05 (Figure 2A).

#### 3.1.4. FB Treatment Caused a Reduction in Water and Food Intake in the Diabetic Animals

Water and food intake were measured daily for animals in the three groups during the treatment period. The water and food intake of the DC group significantly (*p* < 0.05) increased throughout the experimental period compared with that of the NC group. However, water and food intake were significantly lower in the D + FB group compared with the DC group but was significantly higher in the D + FB group compared with the NC group. For all obtained values, the level of significance was *p* < 0.05. The results shown in Figure 2B for water intake and Figure 2C for food intake indicate health improvement in the FB-treated diabetic animals. This improvement is another positive indicator of the benefits of FB treatment for the diabetic animals.

#### 3.1.5. FB Treatment Improved the Physical Appearance of the Diabetic Animals

During the 8-week experiment, the DC animals showed a gradual deterioration in their activities and appearance. The animals were lethargic and slow in movement, while the FB-treated animals showed remarkably improved activities and physical appearance (Figure 2D) comparable to those of the NC animals.

### 3.2. FB Treatment Caused Pancreatic Recovery and the Regeneration of Insulin-Producing β-Cells

#### 3.2.1. Pancreas of FB-Treated Diabetic Animals Produced Insulin and C-Peptide

For the determination of the factors underlying the notable reduction in BG in the FB-treated diabetic animals, insulin was measured in both the serum and pancreatic tissue of the three groups of experimental animals, while the C-peptide level was measured in the sera of the D + FB animals and compared with those in the sera of NC and DC animals.

##### Insulin in Serum

**Figure 3:**
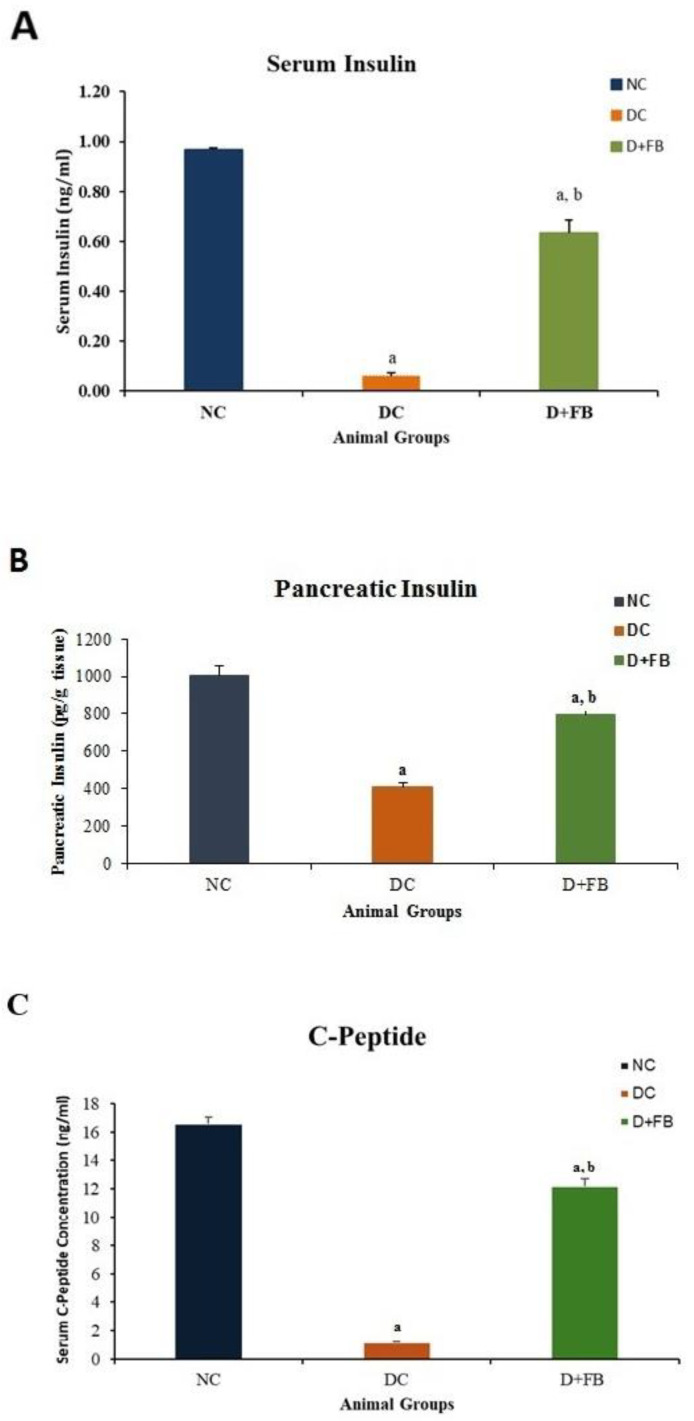
(**A**–**C**) FB treatment resulted in the recovery of the diabetic pancreas and regeneration of insulin-producing β cells. (**A**) A bar graph showing the significant increase in the concentration of insulin in the serum of the FB-treated animals. (**B**) A bar graph showing the concentration of insulin in the pancreas. (**C**) A bar graph showing the concentration of C-peptide in the serum of the D + FB-treated male rats. All are significant (*p* < 0.05) compared to those in the DC and NC rats at the end of 8 weeks of FB treatment.

The serum insulin concentration was determined in each of the three groups of animals. The NC animals had an insulin concentration of 1.036 ± 0.158 ng/mL. The DC group showed a very low level of insulin, 0.052 ± 0.002 ng/mL (5% of that of the NC group, i.e., a 95% reduction), while the FB-treated group showed an insulin concentration of 0.698 ± 0.08 ng/mL, representing 67% of the insulin level in the NC group (i.e., 33% reduction from that in the NC group) and a significant (124%) increase above that in the DC group (Figure 3A). For all obtained values, the level of significance was *p* < 0.05.

##### Insulin in Pancreatic Tissues

Insulin levels in the homogenized pancreatic tissues of animals from each of the three experimental groups were measured. The NC animals had insulin levels of 1003.87 ± 4.634 pg/gm. The diabetic control (DC) animals showed a significant decrease in insulin (409.61 ± 1.213 pg/g; i.e., 41% compared to that in the NC animals), while the D + FB animals had an insulin concentration of 794.63 ± 3.401 pg/g, i.e., 79.2% of the concentration in the NC animals; thus, there was a significant (*p* < 0.05) 21% reduction in insulin levels compared to that of the NC animals. However, the concentration of insulin in the pancreas of the D + FB animals was 794.63 ± 3.401 pg/g (i.e., 70%), greater than that in the pancreas of the DC animals. This was significantly greater (*p* < 0.05) than that in the pancreas of the DC animals (Figure 3B).

##### C-Peptide in Serum

C-peptide levels in the blood sera of the three groups of animals were determined. The C-peptide concentration in the serum of the NC animals was 16.601 ± 0.45 ng/mL, while that in the serum of the DC rats was 1.202 ± 0.03 ng/mL (7.24% of that in the serum of the NC animals). However, the C-peptide concentration in the D + FB rats was 12.193 ± 0.49 ng/mL (73.45% of that in the serum of the NC animals). The C-peptide concentration in the D + FB rats was significantly (ten times) greater than that in the DC group (Figure 3C), confirming the production of insulin by the newly regenerated β cells. For all the obtained results, the level of significance was *p* < 0.05 compared to the DC group.

The presence of significant amounts of insulin in the sera and pancreatic tissue and C-peptide in the sera of the FB-treated diabetic animals indicated the recovery of the islets. The presence of very small amounts of insulin in the serum and pancreas and the presence of a very small amount of C-peptide in the serum of the DC animals indicated that there were traces of active β cells remaining in the pancreas after STZ treatment (Figure 3C).

The bar graphs for the DC animals in Figure 3A–C show that although the islets were destroyed by STZ injection in the DC animals, a small number of active β cells remained in the pancreas and could produce small amounts of insulin and C-peptide.

### 3.3. Comparison of Histopathological Analysis and Immunohistochemical Staining of the Pancreatic Tissues of the Three Groups of Animals

Following the recovery of islet function in the FB-treated diabetic rats, the regeneration of β cells and their insulin production had to be proven. The following experiments were conducted immediately after the animals in the three experimental groups were sacrificed.

#### 3.3.1. Histopathological Analysis of the Pancreatic Tissues of FB-Treated Rats Confirmed Islet Recovery

Histopathological and immunohistochemical (IHC) analyses were performed to evaluate pancreatic islet recovery in STZ-induced diabetes in SD rats treated with FB.

**Figure 4:**
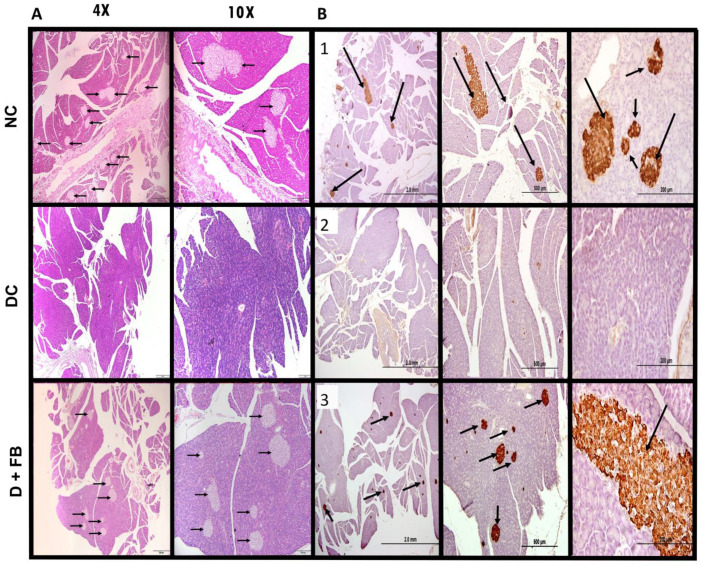
(**A**,**B**): FB treatment resulted in the recovery of structures containing insulin-producing β cells. (**A**) Light microscopy images of pancreatic sections (magnification 4X, 10X). The top two images depict a pancreatic section from an NC animal with black arrows showing intact islets with β cells. The middle two images show a pancreatic section indicating the lack of islets in the pancreas of DC animals. In the bottom two images, the black arrows show several regenerated islets in the FB-treated diabetic animals. (**B**) Light microscopy images of immunohistochemical staining for insulin in pancreatic sections from the NC, DC, and FB-treated diabetic rats at different magnifications 4X, 10X, 40X (scale bars for panel 1: 2.0 mm, panel 2: 500 µm, panel 3: 200 µm). The top images {1} show a pancreatic section from an NC animal with arrows pointing at the insulin immunostained islets. Middle images {2} show a lack of islets in the pancreatic section of the STZ-treated diabetic rats. Bottom images {3} depict arrows pointing at the regenerated islets in the pancreatic section of the D + FB-treated rats, which show immunoactivity for insulin, thus confirming the newly developed structures to be islets of Langerhans and the regeneration of the insulin-producing β cells.

Histological examination (Figure 4A, top section) by light microscopy revealed intact islets of Langerhans in the pancreases of the NC animals. In contrast, the pancreatic tissues of the DC group showed no islets of Langerhans (Figure 4A, middle section). However, the FB-treated diabetic animals exhibited islets with morphologies resembling those of the normal control (NC) group, as indicated by the black arrows (Figure 4A, bottom section), indicating structural regeneration.

#### 3.3.2. Immunohistochemical Staining for Insulin in the Recovered Islets of the Pancreases of the FB-Treated Diabetic Animals Showed Insulin in β-Cells

For confirmation that the recovered islets contained insulin-producing β-cells, pancreatic tissues from the three groups of sacrificed animals were processed for insulin immunohistochemical studies. Sections of the pancreas from each immunostained group were examined by light microscopy. The pancreatic tissues of the NC animals {1} showed normal insulin immunoreactivity in the islets of Langerhans, as indicated by the black arrows. The pancreatic tissues of the DC group {2} showed no islets and no clear insulin-immunoreactive β-cells. However, pancreatic tissues from the D + FB-treated animals {3} showed insulin-immunostained β-cells in the regenerated structures after FB treatment. The structures contain insulin-producing β-cells, as indicated by the black arrows (Figure 4B {3}). These findings demonstrate that FB treatment induces the regeneration of functional islets capable of insulin production. While these results confirm β-cell recovery, further characterization using multi-hormone immunofluorescence is needed to fully assess the endocrine composition of the regenerated islets.

### 3.4. FB Treatment Improved Health Issues Related to Diabetes

Diabetes causes certain health issues, including oxidative stress, inflammation, and alteration in the lipid profiles. Each of these three concerns is a serious health problem that needs to be addressed. Any procedure that alleviates any or all these issues will lead to improvements in the general health of diabetic patients. Experiments were conducted to evaluate the effect of FB treatment on oxidative stress, inflammation, and the lipid profiles in the treated diabetic animals.

#### 3.4.1. FB Treatment Optimized Oxidation/Antioxidation in the Kidneys and Liver of Diabetic Male Rats

Oxidative stress in diabetic patients manifests in the kidneys and liver. The antioxidant components in FB, such as F-acids, cholesta-3,5-diene, and IL-19, were predicted to reduce oxidative stress in FB-treated animals. The following results were obtained for the effect of FB on oxidative stress.

##### FB Treatment Reduced MDA Concentration in Both the Kidneys and Liver

**Figure 5:**
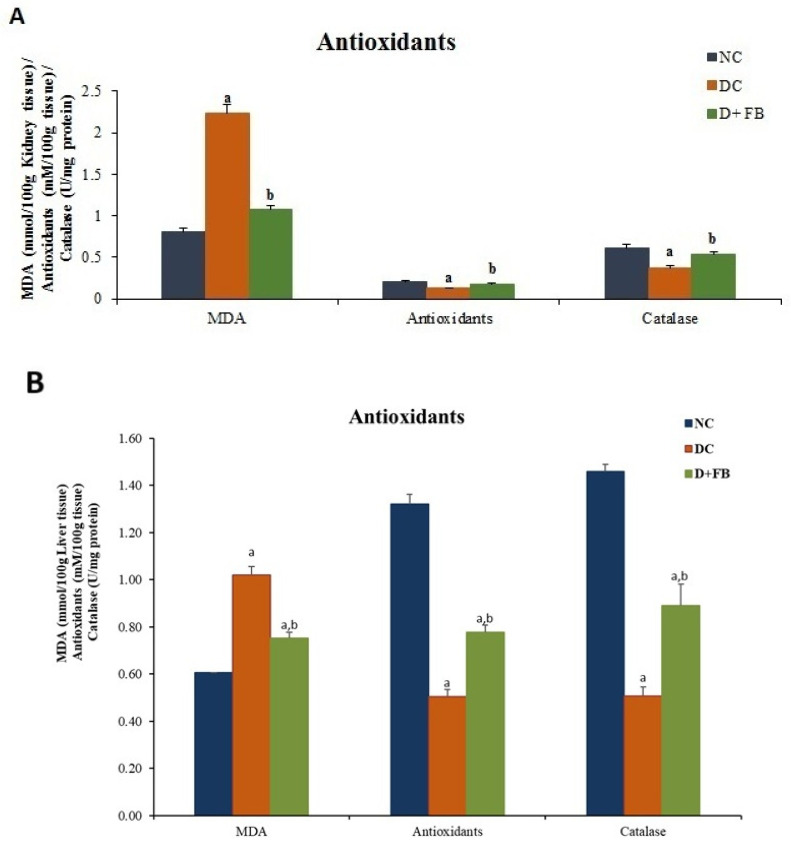
(**A**,**B**) FB treatment improved the antioxidant profiles in the kidney and the liver of the diabetic rats. (**A**) Bar graph showing the benefit of FB treatment on kidney antioxidant parameters and (**B**) bar graph showing the benefit of FB treatment on liver antioxidant parameters in the serum of the D + FB-treated male rats. Liver antioxidant parameters, such as MDA, antioxidant, and catalase levels, are significant (*p* < 0.05) compared to those in the DC and NC rats at the end of 8 weeks of FB treatment. However, kidney antioxidant parameters, such as MDA, antioxidant, and catalase levels, were significant (*p* < 0.05) compared to those in the DC rats at the end of 8 weeks of FB but were not significantly different from those of NC rats.

The kidney: The kidneys of the DC group had MDA concentrations 174.97% higher than those of the NC group, while the MDA concentration in the D + FB group was 31.94% higher than that in the NC group. The FB-treated animals showed a significant reduction in the kidneys’ MDA level at 52% compared with that of the DC animals (Figure 5A). The MDA level was significantly lower in the kidneys of FB-treated diabetic animals by 143.06% compared with that in the kidneys of the DC animals.

The liver: The livers of the DC group had MDA concentrations 67.76% higher than those of the NC group, while the MDA concentration in the D + FB group was 23.68% higher than that in the NC group. The MDA concentration in the liver of the D + FB group was significantly lower by 26% compared with that in the DC group (Figure 5B). The MDA level was significantly lower by 44.07% in the livers of FB-treated diabetic animals compared with those in the livers of DC animals. All the results are significantly different (*p* < 0.05) for both the kidneys and livers of the FB-treated animals compared with those of the DC animals.

##### FB Treatment Increased the Concentration of Antioxidants in Both the Kidneys and Liver

The kidneys: The antioxidant concentration in the kidneys was 39% lower in the DC group compared with that in the NC group, while the antioxidant concentration in the D + FB group was 14% lower than that in the NC group. The antioxidant level increased significantly by 40% in the kidneys of FB-treated diabetic animals compared with that in the kidneys of DC animals (Figure 5A).

The liver: The antioxidant concentration in the livers of the D + FB animals was significantly greater by 54% than that in the livers of the DC animals (Figure 5B). The livers of the DC group had an antioxidant concentration 61.23% lower than that in the NC group, while the antioxidant concentration in the D + FB group was 40.15% lower than that in the NC group.

All the results are significantly different (*p* < 0.05) for both the kidneys and livers of FB-treated animals compared to those in the DC group.

##### FB Treatment Increased Catalase Concentration in Both the Kidneys and Liver

The kidneys: The catalase concentration in the kidneys of the DC group was 39% lower than that in the NC group. Moreover, the catalase concentration in the D + FB group was 13% lower than that in the NC group. In the FB-treated diabetic animals, the catalase level increased significantly by 44% in the kidneys, compared with that in the kidneys of the DC animals (Figure 5A).

The liver: The catalase concentration in the livers of the DC group was 65.27% lower than that in the NC group, while the catalase concentration in the D + FB group was 39.04% lower than that in the NC group. However, compared with that in the DC group, the liver of the D + FB-treated animals showed a 76% increase in catalase concentration (Figure 5B). All the results are significantly different (*p* < 0.05) for both the kidneys and livers from FB-treated animals compared with those in the DC group.

These results show that FB treatment optimizes oxidation/antioxidation levels in the kidneys and livers of FB-treated diabetic animals; thus, FB treatment improves oxidative stress in diabetic animals, hence contributing to their recovery. Accordingly, the translational research for the improvement of oxidative stress in humans using FB treatment will be worthwhile.

### 3.5. FB Treatment Reduced the Proinflammatory Cytokine and Elevated the Anti-Inflammatory Cytokine Levels

Blood sera from each of the three experimental groups were analyzed for the levels of the proinflammatory cytokines IL1α, IL1β, IL4, IL6, IL12, IL13, TNFα, GMC-SF, and RANTES, and the anti-inflammatory cytokines IL2, IFNγ, and IL10. 

#### 3.5.1. FB Treatment Reduced the Levels of Proinflammatory Cytokines

**Figure 6:**
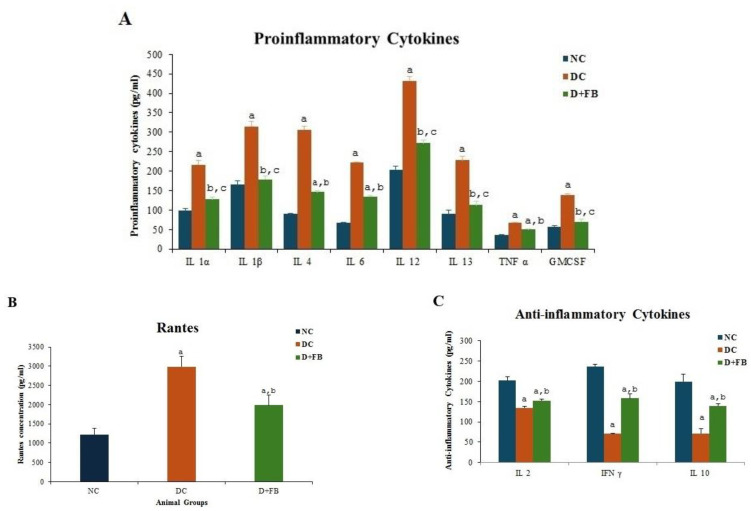
(**A**–**C**) FB treatment caused a reduction in proinflammatory cytokine and RANTES levels and an increase in anti-inflammatory cytokine levels in the serum of the treated diabetic animals. (**A**) Bar graph showing the benefit of FB treatment on proinflammatory cytokine levels. (**B**) Bar graph showing RANTES levels. (**C**) Bar graph showing anti-inflammatory cytokine levels in the serum of the D + FB-treated male rats. Proinflammatory cytokine and RANTES levels showed a significant (*p* < 0.05) reduction whereas anti-inflammatory cytokines showed a significant increase in level compared to those in the DC and NC rats at the end of 8 weeks of FB treatment.

First, the proinflammatory cytokines (IL1α, IL1β, IL4, IL6, IL12, IL13, TNFα, and GMC-SF) were evaluated. Compared with those in the NC group, the concentrations of all proinflammatory cytokines in the DC group were significantly greater. However, FB treatment significantly decreased the concentrations of all proinflammatory cytokines in diabetic animals compared to those in the untreated diabetic animals (Figure 6A).

#### 3.5.2. FB Treatment Reduced the Levels of Proinflammatory Cytokine RANTES

Additionally, an examination of the serum of the DC animals revealed that the level of the proinflammatory cytokine RANTES was significantly higher in DC animals than in the NC animals. However, compared with the untreated DC animals, FB treatment led to a significant decrease in the level of the proinflammatory cytokine RANTES in diabetic animals (Figure 6B).

#### 3.5.3. FB Treatment Elevated the Anti-Inflammatory Cytokine Levels

The levels of anti-inflammatory cytokines (IL2, IFNγ, and IL10) in the DC animals were significantly lower than those in the NC animals. However, the FB-treated animals showed significant increases in the levels of the anti-inflammatory cytokines IL2, IFNγ, and IL10 compared with those in the DC group (Figure 6C).

All the results were significantly different in terms of the proinflammatory, anti-inflammatory cytokine, and RANTES levels (*p* < 0.05) in the FB-treated animals compared with those in the DC animals. FB treatment resulted in a reduction in the levels of proinflammatory cytokines and an increase in the levels of anti-inflammatory cytokines in diabetic animals. This is an added benefit in addition to the other advantages of FB treatment that might help accelerate the recovery of diabetic animals. The translational research into the application of FB treatment for the reduction in inflammation in patients suffering from diseases that cause inflammation will be a desirable step.

### 3.6. FB Treatment Improved Lipid Profiles

FB treatment of diabetic animals improved the lipid profiles, as shown in the following subsections.

#### 3.6.1. FB Treatment Decreased Serum Cholesterol

**Figure 7:**
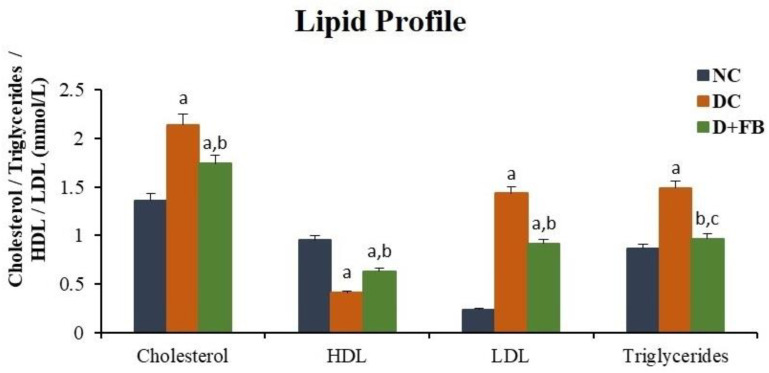
FB treatment improved the serum lipid profiles of diabetic animals. The bar graphs show significant (*p* < 0.05) improvements in the serum lipid concentrations of cholesterol, HDL, LDL, and triglycerides in the D + FB-treated animals compared with those of the DC animals. This is an added benefit of FB treatment.

The concentrations of cholesterol in the blood sera from each of the three groups of male animals were measured. The NC animals had a cholesterol concentration of 1.36 ± 0.108 mmol/L, while the cholesterol concentration in the DC animals increased by 57% compared with that of the NC animals (2.14 ± 0.122 mmol/L). However, the cholesterol level in the FB-treated diabetic animals was 28% greater than that in the NC animals (1.74 ± 0.087 mmol/L) and 18.7% less than that in the DC animals (Figure 7). All the results are significantly different (*p* < 0.05) for the NC and D + FB animals compared with those for the DC animals.

#### 3.6.2. FB Treatment Increased HDL in Serum

The HDL concentrations in the blood sera from each of the three groups of male rats were measured and compared with those in the NC group. The HDL concentration in the NC group was 0.95 ± 0.077 mmol/L, while the HDL level in the DC group decreased by 57% (0.41 ± 0.030 mmol/L). However, the D + FB group had an HDL concentration of 0.63 ± 0.022 mmol/L, which is a 34% reduction compared with that in the NC group, but a significant 54% increase compared to that in the DC group (Figure 7). This result indicates the beneficial effect of FB treatment in increasing the HDL level in the diabetic FB-treated animals. All the results are significantly different (*p* < 0.05) for the NC and D + FB animals compared with those of the DC animals.

#### 3.6.3. FB Treatment Decreased LDL in Serum

The sera LDL concentrations were calculated for all three experimental groups. The concentration of LDL in the NC animals was 0.237 ± 0.007 mmol/L, while that in the DC group was 1.432 ± 0.037 mmol/L, a significant increase of 504%. The D + FB group had an LDL serum concentration of 0.917 ± 0.017 mmol/L LDL, representing an increase of 287% compared with the NC group, but a significant 36% reduction compared to that in the DC group (Figure 7). This result reflects the beneficial effect of FB treatment in lowering the LDL level in the treated diabetic animals. All the results were significantly different (*p* < 0.05) for the NC and D + FB animals compared with those of the DC animals.

#### 3.6.4. FB Treatment Decreased Triglycerides in Serum

The concentrations of triglycerides in the blood sera from each of the three groups of animals were measured. The NC animals showed a triglyceride concentration of 0.869 ± 0.056 mmol/L, while the level of triglycerides in the DC group was 1.49 ± 0.063 mmol/L, a 71% increase compared with the NC animals. However, the D + FB group showed a significant (35%) reduction in the triglyceride concentration (0.968 ± 0.028 mmol/L) compared to that in the DC group. Notably, the triglyceride concentration in the FB-treated animals was only 11% greater than that in the NC animals (Figure 7). All the results are significantly different (*p* < 0.05) for the NC and D + FB animals compared with those of the DC animals.

FB treatment reduced cholesterol, LDL, and triglyceride concentrations, and elevated HDL concentration in serum. These changes were expected to contribute to improvements in the general health of the FB-treated animals, thus aiding in the recovery of the diabetic animals. The improvements in the lipid profiles after FB treatment may not be restricted to diabetic cases, but FB may be applied to treat other urgent health issues in humans where the lipid profiles are altered. This can only be proved by the translational research into the beneficial effects of FB treatment on other health issues that are accompanied by changes in the lipid profiles.

### 3.7. FB Treatment Did Not Cause Side Effects in Normal Animals After 16 Weeks of Treatment

**Figure 8:**
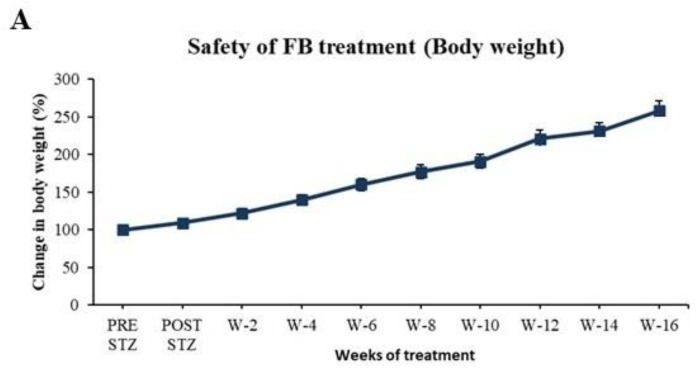

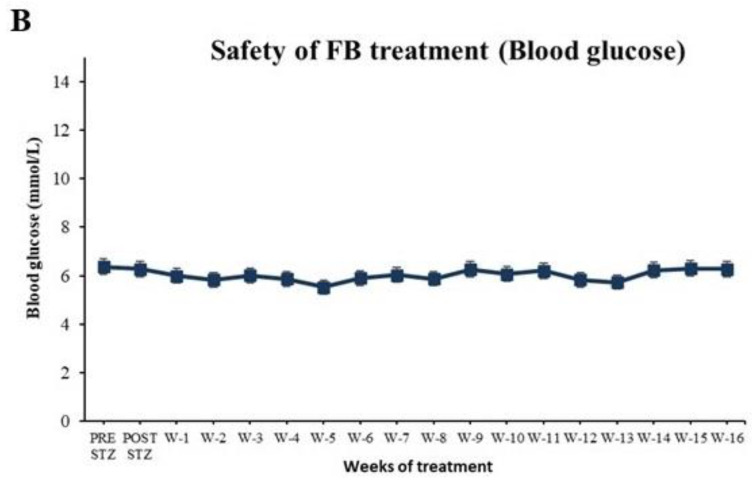
(**A**,**B**) Shows the safety of FB treatment for male NC rats treated for 16 weeks instead of the 8-week standard period. (**A**) The unhindered growth was shown in the increase in body weight in the FB-treated animals. (**B**) Safety was also shown by stable blood glucose levels.

To evaluate the safety of FB treatment, normal non-diabetic (NC) SD male rats were subjected to IP administration of 3 mg FB/kg body weight for 16 weeks instead of 8 weeks. The treated animals appeared healthy and grew normally in terms of size and weight, with an average increase in weight from 180 g to 330 g (Figure 8A), which was comparable with that in the normal untreated group (Figure 2A). Moreover, the BG levels of the FB-treated animals were within the normal range throughout the experiment (Figure 8B) and comparable with those in the NC group (Figure 1A). After 16 weeks, the animals were sacrificed under anesthesia, and their organs were examined for gross pathological changes. No deformities or gross pathological alterations were found in any of the organs.

### 3.8. Regenerated β Cells Remained Active for 48 Weeks Until the Animals Were Sacrificed

After the treatment of the diabetic male animals with FB for 8 weeks, the treatment was discontinued, and the animals were evaluated for 40 weeks. The animals were then sacrificed and evaluated for the action of the regenerated β cells on body weight, BG level, serum insulin, and C-peptide.

#### 3.8.1. Body Weight

**Figure 9:**
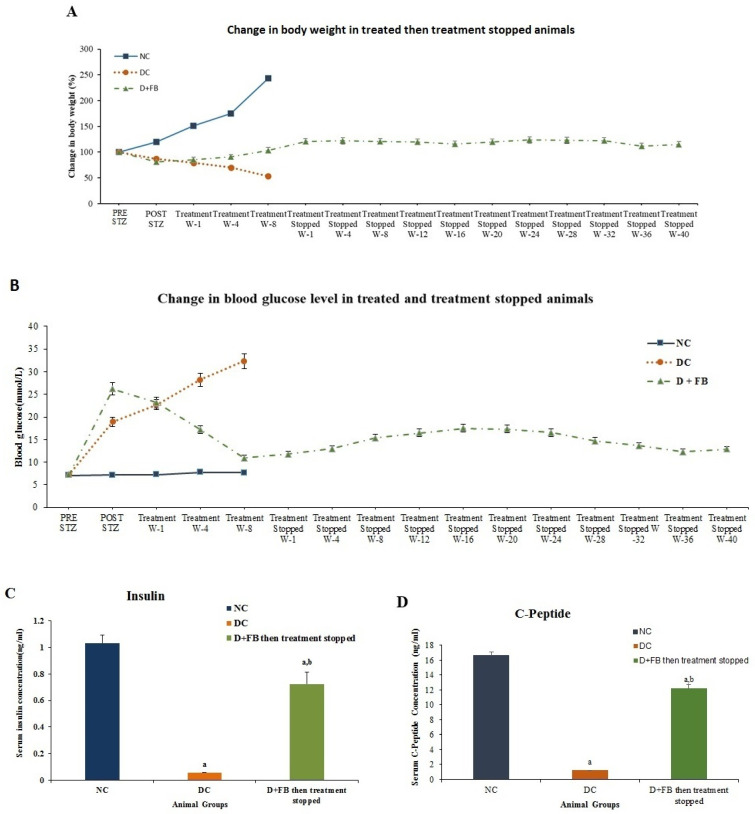
(**A**–**D**) Shows the durability of FB treatment. Diabetic animals were treated for 8 weeks. At the end of the 8 weeks their treatment was discontinued, and the animals were monitored for 40 weeks. (**A**) A line graph shows the increased body weight of the FB-treated diabetic animals over the 48-week period compared to the diabetic nontreated animals. (**B**) A line graph showing the long-lasting reduction in BG level in the FB treated animals compared to that of the DC animals, indicating the durability of the activity of the regenerated β cells in insulin production. Bar graphs show insulin (**C**) and C-peptide (**D**) production in the blood serum of the FB treated diabetic animals compared with those of the DC animals. All these events took place in the FB-treated diabetic animals during 48 weeks without the use of immunosuppressants. The results for the 8 week FB-treated diabetic animals then their treatment was discontinued for 40 weeks period during which the animals were monitored, and those of the DC animals are significant (*p* < 0.05) compared with those of the NC animals.

The DC group showed a gradual decrease in body weight, and the NC group showed an increase in body weight until the two groups were sacrificed. The weights of the FB-treated animals for 8 weeks then their treatment was discontinued for 40 weeks stayed almost steady throughout the 48-week experimental period (Figure 9A).

#### 3.8.2. Reduction in BG Levels in the FB-Treated Diabetic Animals for 8 Weeks. Their Treatment Was Then Discontinued and the Animals were Monitored for 40 Weeks

In the FB-treated diabetic animals for 8 weeks then the treatment was discontinued, and the animals were monitored for 40 weeks, the BG gradually decreased to a level close to those of the normal control rats (Figure 9B), while those of the DC group notably increased. The BG levels of the 8-week FB-treated animals then the treatment was discontinued indicated that the newly regenerated β cells remained active for 48 weeks until the animals were sacrificed. These β cells were not attacked by the immune system. No immunosuppressants were used in these experiments. These results indicate that FB treatment provides benefits that are not matched by the other two procedures used in the research to combat diabetes.

#### 3.8.3. Increased Serum Insulin Concentration in the 8 Week Treated Animals Then the Treatment Was Discontinued for 40 Weeks

The serum insulin concentration was measured in the three groups (NC, DC, D + FB) of animals. The D + FB group was the 8-weeks treated, then their treatment was discontinued and was then monitored for 40 weeks. The serum insulin concentration was found to be 1.033 ng/mL in the NC group and 0.056 ng/mL in the DC group, i.e., 5.4% of that in the NC group. However, the concentration was 0.725 ng/mL in the D + FB-treated group after 40 weeks of discontinued FB treatment, i.e., 70% of the insulin in the NC animals. This was significantly higher than that in the DC group (1194.64%, *p* < 0.05) (Figure 9C).

#### 3.8.4. Increased C-Peptide in the 8-Week Treated Animals Then Their Treatment Was Discontinued for 40 Weeks

The C-peptide levels in the blood serum of the three groups (the NC, the DC, and the 8 weeks D + FB treated then their treatment was discontinued and were evaluated for 40 weeks) were measured. C-peptide levels in the serum of the D + FB-treated were compared with those in the serum of the NC and DC animals. The concentration of C-peptide in the NC animals was 16.5667 ± 0.9333 ng/mL and 1.202 ± 0.029 ng/mL in the DC animals (7% of that in the NC animals), while in the D + FB-treated animals, the concentration was 12.1933 ± 0.49 ng/mL (74% of that in the NC animals). The results for the D + FB-treated animals and the DC animals were significant (*p* < 0.05) compared to those of the NC animals (Figure 9D).

### 3.9. FB Contains Growth Factors and Biologically Active Proteins That Can Play Roles in the Regeneration of β Cells

This study searched for growth factors and active proteins in FB that are homologous to and resemble those in rats and humans using a specific ELISA kit for each protein. Accordingly, those that have been found are called rat and human growth factors for ease of remembrance. Eighty-one growth factors and biologically active proteins, including stem cell factors that can play roles in β-cell regeneration and contribute to wound healing and improvements in diabetes, were discovered in FB. Each was listed with its ELISA kit and a reference indicating its known biological activity in Table 1.

## 4. Speculated FB Action in Pancreatic Recovery

**Figure 10:**
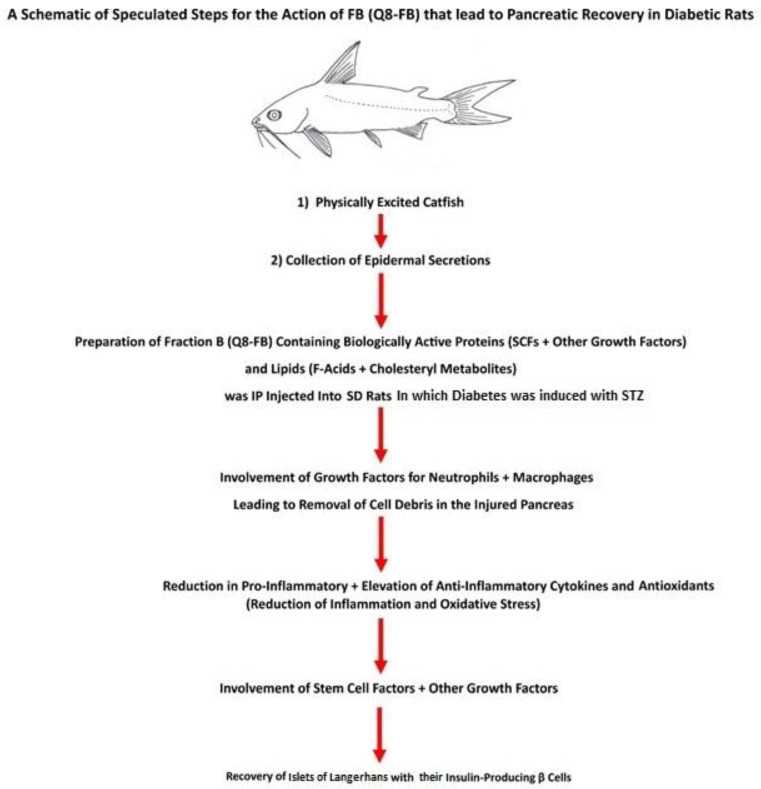
A schematic of the proposed action of FB in pancreatic recovery in diabetic SD rats.

Our first hypothesis presumed that the potent wound healing FB could cure the injured diabetic pancreas after STZ injection and could cause the regeneration of the β-cells. Our results prove this hypothesis to be valid. Our second hypothesis assumed the involvement of growth factors that might be present in FB in the recovery of the diabetic pancreas and the regeneration of β-cells. Remarkably, our hypothesis was ascertained by discovering a total of 81 growth factors and active proteins, including stem cell growth factors, whose activities are related to improving diabetes, supporting wound healing, and contributing to regeneration. They are shown in Table 1. It is speculated that the islets may have recovered, and the insulin-producing β-cells may have been regenerated in FB-treated diabetic animals due to the action of the active protein components in FB, especially the stem cell factors aided by the active lipids through their synergistic and cooperative action. A speculative interpretation of the events that led to β-cell regeneration is shown in a schematic in Figure 10 depicting the steps that are proposed to lead to the regeneration of β-cells in T1D SD rats after FB treatment. This speculation depends on the assumptions stated in our first and second hypotheses. It is possible that the growth factors in FB, especially the stem cell factors and the active lipids in FB, would facilitate the recovery of the diabetic pancreas. As the growth factors and active proteins are present in FB in minute quantities, they might not act according to their known roles but will act as growth factors to promote the recovery of the injured pancreas and regeneration of active β-cells. The following two examples support this speculation:

1. Among the active proteins found in FB is insulin, which was detected at a concentration of 139.597 ± 3.504 ng/g FB protein, which is equal to an injection of 0.08378 ng insulin/200 g body weight in this study. The literature shows that the daily insulin requirement of a diabetic rat is in the range of 0.1–0.4 units (equivalent to a range of 3470–13,880 ng) per 200 g of animal body weight. The amount of insulin injected via FB into diabetic rats is minute compared to the daily insulin requirement; hence, its effect on BG level is negligible.
2. FB also contains glucagon at a concentration of 13.581 ± 0.3726 ng/g FB protein. The amount of FB injected into the diabetic rat contained 0.008149 ng glucagon/200 g rat body weight each day. This concentration is very small compared to the insulin concentration required daily by the rat. Accordingly, neither of these two exogenous proteins, insulin and glucagon, were present at high enough concentrations to influence the BG levels in FB-treated rats. This conclusion was supported by the fact that when the diabetic animals were treated with FB for 8 weeks then their treatment was suspended for 40 weeks no elevation in BG levels in the FB-treated experimental animals was observed during the 40 weeks of suspended FB treatment until the animals were sacrificed. Suspending FB injection for 40 weeks resulted in the animals being deprived of these two exogenous proteins (insulin and glucagon). This finding also confirms the lack of effect of both exogenous proteins on the BG levels in FB-treated animals.

FB was found to contain human insulin (HI), rat insulin (RI), human insulin-like growth factor-1 (HIGF-1), rat insulin-like growth factor-1 receptor (RIGF-1), human and rat glucagon-like peptide-1 (HGLP-1), and rat glucagon (RGCG) in minute concentrations. Normally, the activity of each of these components influences glucose levels in the blood, if present in high enough concentration, but they exist in very small concentrations. Also, the mode of action of each is different from the others; therefore, they cannot act in unison to provide a one-directional change in glucose levels at any given time. Our third hypothesis proposed that the roles of these compounds are not to influence the BG levels in FB-treated diabetic animals, as they are normally expected to do, but to contribute to the regeneration of β-cells, by acting as growth factors.

3. It was speculated that the HI, RI, HIGF-1, RIGF-1, HGLP-1, and RGCG found in FB in minute concentrations were components of the total aggregated active proteins and growth factors, whose combined action might have contributed to healing the injured pancreas and islet recovery, as was shown by the regeneration of insulin-producing β-cells. It has been well-established that insulin supports wound healing and that GLP-1 promotes β-cell proliferation through mitogenesis, while decreasing β-cell apoptosis [1,4,49,50,51]. The quantitative data presented in this study supports our hypothesis, which presumed that FB would be able to heal the STZ-injured [90] diabetic pancreas.

Our research results indicate that several specific growth factors and active proteins, including stem cell factors and specific active lipids, are required to work in a synergistic and cooperative manner for the successful recovery of Islets and regeneration of active β-cells in the diabetic pancreas. These growth factors and active lipids were found to exist in FB.

## 5. Discussion

FB treatment led to the recovery of one of the most important functions of the pancreas, namely the control of BG level, and its reduction to near that of the NC animals. The five-times-repeated experiment (three times in treating male diabetic SD rats, one time in treating diabetic animals for 8 weeks then their treatment was suspended for 40 weeks experiment, and one time in treating female diabetic SD rats) proved the efficacy of FB treatment in male and female diabetic SD rats.

Our measurement of significant levels of insulin in serum and pancreatic insulin in the FB-treated diabetic animals point to the regeneration of insulin producing β-cells. The pancreatic insulin content in homogenized tissue provided a robust assessment of total insulin reserves, confirming significant β-cell recovery in FB-treated rats (Figure 3B). While homogenization ensured comprehensive insulin recovery, islet isolation could offer complementary insights into islet-specific insulin production and cellular composition. This approach, though technically challenging and prone to insulin loss during processing, may further validate the functionality of regenerated islets. Future studies will incorporate islet isolation and multi-hormone analyses to characterize the endocrine profile of FB-recovered islets, building on the promising regenerative effects observed here.

Histopathological and immunohistochemical (IHC) analyses confirmed that FB treatment induced the regeneration of functional islets in the STZ-induced T1D rat model. The restored islet architecture (Figure 4A) and strong insulin positivity in IHC staining (Figure 4B) demonstrate significant β-cell recovery, supporting FB’s regenerative potential for T1D treatment. However, while these data indicate the presence of functional islets capable of insulin production, further characterization is needed to confirm their complete endocrine composition. Immunofluorescence (IF) analysis for multiple hormones (e.g., glucagon, somatostatin, and pancreatic polypeptide) would provide a comprehensive assessment of whether the regenerated islets fully resemble normal islets with all endocrine cell types. Due to resource constraints, we prioritized insulin-specific IHC in this study, but future studies will employ multi-hormone IF to validate the endocrine profile of FB-induced islets, building on the promising results reported here.

The presence of minute amounts of HI, RI, HIGF-1, RIGF-1, HGLP-1, and RGCG as exogenous compounds in FB do not explain the recovery of the pancreatic function, as manifested in insulin production by the regenerated insulin-producing β-cells. After all, patients who regularly take insulin to control their BG still suffer from the lack of increase in their active β-cells and deterioration and malfunction of their vital organs and tissues. The administration of insulin only seems to slow this deterioration.

Although the mechanism of action of the collective active component (especially the 81 growth factors) in FB in the recovery of the diabetic pancreas is unknown, their known individual intrinsic activities cannot be ignored. The literature indicates that Substance-P in combination with epidermal stem cells promotes wound healing and nerve regeneration [48]. Fraction B was found to contain Substance-P, stem cell factors, nerve growth factor, and vascular endothelial growth factor, among other factors. These components amongst other wound healing components, such as FGF1, FGF19, FGF21, EGFs, EPOs, PDGF, and RCTGF, and growth hormone [39,40,41,42,43,86,87,88,89] in FB were speculated to play major roles in the recovery of the injured pancreas and the improvement of diabetic symptoms.

Macrophages have been reported to be the first cells to invade the islets after the repeated administration of STZ [90]. The presence of macrophage colony-stimulating factors in FB might lead to an increase in the number of indigenous macrophages through regeneration. The increase in neutrophils and macrophages in FB-treated animal wounds [10,37,44,90] supported the proposed role of macrophages in the recovery of pancreatic function in FB-treated diabetic animals. This effect could accelerate the clearing of cell debris in the injured diabetic pancreas, thus paving the way for recovery.

The presence of neutrophil activating protein-2 (NAP-2) in FB helps explain the high number of neutrophils found in FB-treated wounds and the observed antibiotic action of FB. Here, the antibiotic action might be due to NETosis caused by the action of F6 and the other F-acids, as well as some of the oxysterols found in FB on neutrophils. Moreover, the presence of NAP-2 in FB might have supported the recovery of FB-treated diabetic pancreases and enhanced the regeneration of β cells. NETosis following FB treatment can have several applications in human health [10,91]. Stem cell factors found in FB might have led to the multiplication of stem cells in the diabetic pancreas. In turn, the newly regenerated stem cells could have played a major role in the observed recovery of the diabetic pancreas. C-peptide concentration in the blood serum of the FB-treated diabetic animals reflects the activities of the regenerated β-cells. The C-peptide is a biologically active protein whose activities span over several biological activities to include binding to cell-membrane surface-signaling molecules and the activation of downstream signaling pathways to act as antioxidant, anti-inflammatory, and anti-apoptotic complex structures, or regulate cellular transcription through internalization [85]. According to C-peptide concentration, it can play an important role in diabetic symptoms. The concentration of C-peptide found in the blood serum of normal control (non-diabetic) animals and in the blood serum of FB-treated diabetic animals was much higher than the concentration of insulin found in the blood serum of both groups of animals. This higher concentration of C-peptide may reflect the good health status of both groups of animals.

FB treatment also provided exceptional benefits to diabetic animals. These benefits could be attributed to the activities of the anti-inflammatory components (IL19, IL37, IL-11, F-acids, C-peptide, and S5) and the antioxidants (F-acids, cholesta-3,5-diene, IL-11, IL19, and C-peptide) found in FB. FB treatment provided a third important benefit. It improved lipid profiles. The factors that led to the improvement in the lipid profiles remain to be determined. These added benefits are anticipated to improve the general health of the FB-treated animals, thus supporting pancreatic recovery and β-cell regeneration.

Remarkably, the 8-week FB treatment caused the regenerated islets to produce insulin for 48 weeks until the animals were sacrificed. Furthermore, the regenerated insulin-producing β-cells in this study were not destroyed by the immune system of the FB-treated animals. This supports the speculation that the newly regenerated β-cells were indigenous in origin, whether they originated from the residual β-cells in the diabetic pancreas or through the conversion of the other pancreatic cells into β-cells. FB treatment did not require the use of immunosuppressants. This represents a major advantage over the other two procedures, namely, the β-cell and islet transplantation procedure and the stem cell procedure, where it is necessary to employ immunosuppressants, despite their costs and negative side effects. This interesting finding supports future translational research for the application of FB for the treatment of diabetes, as no immunosuppressants will be required to accompany the treatment.

The recovery of the pancreas in the FB-treated diabetic animals and the regeneration of β-cells confirmed our first and second hypotheses that it would be possible to treat the injured diabetic pancreas with FB and regenerate active β-cells. FB seems to contain multiple components having multiple functions that are required for the recovery of the pancreas and regeneration of insulin-producing β-cells in a diabetic animal model. Hence, the remarkable but puzzling effects of FB treatment on the diabetic pancreas can be understood.

Histopathological and immunohistochemical (IHC) analyses confirmed that FB treatment induced the regeneration of functional islets in the streptozotocin (STZ)-induced T1D rat model. The restored islet architecture (Figure 4A) and strong insulin positivity in IHC staining (Figure 4B) demonstrate significant β-cell recovery, supporting FB’s regenerative potential for T1D treatment. However, while these data indicate the presence of functional islets capable of insulin production, further characterization is needed to confirm their complete endocrine composition. Immunofluorescence (IF) analysis for multiple hormones (e.g., glucagon, somatostatin, and pancreatic polypeptide) would provide a comprehensive assessment of whether the regenerated islets fully resemble normal islets with all endocrine cell types. Due to resource constraints, we prioritized insulin-specific IHC in this study, but future studies will employ multi-hormone IF to validate the endocrine profile of FB-induced islets, building on the promising results reported here. We acknowledge the value of GTT for assessing islet function. The current data (BG, insulin, C-peptide, and IHC) provide robust evidence of functional β-cell regeneration. However, performing a GTT test would enhance the characterization of the regenerated β-cell. This test will be attempted in our future studies to validate the glucose responsiveness of the regenerated β-cells.

Our results indicate the presence of some residual β-cells in the pancreas after STZ treatment (Figure 3A–C). There are several possibilities for the regeneration of β-cells in the FB-treated animals to have taken place. Whether the regeneration resulted from the action of the growth factors and active lipids present in FB or from multiplication caused by stem cells of traces of β cells left in the pancreas after STZ treatment, or from the transformation of other pancreatic cells (α-, δ-, or ε) into β cells, as reported to be theoretically possible [1], is an important point worth investigating. It is feasible that the regeneration process in our studies took place through the multiplication of the residual β-cells left after STZ treatment, although the possibility of the conversion of the other pancreatic cells into β-cells at the same time should not be excluded. It will be interesting to determine whether FB will act on human T1D pancreases in the same way it acted on the rat T1D pancreases. If this treatment is effective in human T1D, then human T2D is expected to be easily addressed, as it contains a higher number of active β-cells. Our future studies will investigate this crucial point.

The safety aspects of FB were assessed when normal SD rats were injected daily with FB for 16 weeks. The rats grew normally, and their BG was normal (Figure 8A,B). The animals showed no toxicity or side effects. In separate studies involving IP injection of FB for the treatment of crushed sciatic nerves and pancreatic cancer in animals, no toxicity or side effects were observed [15,16], thus supporting the safety of FB application. These results encourage the consideration of FB for the translational research into the treatment of diabetes.

The mode of action of each of the several growth factors and active proteins, especially stem cell factors, along with the active lipids are well described in the literature. Their presence in FB played important roles in the protection and recovery of the pancreas in FB-treated diabetic animals. It is speculated that the complex, cooperative, and synergistic action of these active components in FB resulted in the observed unmatched recovery of the injured diabetic pancreas. The health benefits achieved with FB treatment are likely not to be attributable to a single component in FB, as no single component in FB can achieve all these results. This study was a departure from the research relying on the action of one single component, as no single component in FB or elsewhere could achieve what was achieved with the FB composition. The incomplete success of all the research efforts undertaken by others to regenerate durable insulin-producing β-cells without ramifications may be due to the absence of essential growth factors and other active components required for the regeneration of β-cells in their studies.

FB and its components may be considered a third arm, in addition to the recent stem cell research and β-cell and islet transplantation in the effort to combat diabetes and its complications. Our results fill the gaps left by the other two procedures. The unmatched benefits of FB treatment, the present high treatment costs, and the health risks involved in diabetes treatment employing the other two procedures make FB treatment attractive for the translational research into the treatment of diabetes, as well as for the translational research into the treatment of diseases that involve inflammation, oxidation stress, and alteration in the lipid profiles, and those that require regeneration to heal. Regeneration of β-cells was reported to be the future goal for curing diabetes that has no cure [4]. Regeneration through FB treatment provides support for such hope.

## 6. Conclusions

Our study results proved that it is possible to heal the injured pancreas in the STZ-induced diabetes in SD rats and cause the regeneration of insulin producing β-cells with FB treatment. This procedure fills the gaps left by the other two procedures used in research to combat diabetes, namely the islet and pancreatic transplantation procedure and the stem cell procedure. The regenerated β-cells in our procedure are long-lasting. They stayed active for 48 weeks until the animals were sacrificed. The regenerated β-cells were not destroyed by the immune system of the animals. No immunosuppressants were used during the experiments. This could mean that the regenerated β-cells were recognized by the immune system as indigenous cells. Other advantages of our procedure over the other two procedures are improvement of health issues related to diabetes. These are reduction of inflammation and oxidation stress and improvement of the lipid profile in the treated diabetic animals. No toxic or side effects were detected with the use of FB in the treatment of the diabetic animals. These advantages improved the health environment of the treated diabetic animals and facilitated the recovery of the diabetic pancreas and the regeneration of the insulin producing β-cells. If the successful treatment of T1D in the SD animal model can be repeated in human T1D patients, then it is expected to be useful in treating T2D, as T2D contains more β-cells than those in T1D in the pancreas. These results make it attractive to use FB for translational research into treatment of diabetes.

## Figures and Tables

**Table 1:** Details of the ELISA kits used for the assay of growth factors and biologically active proteins in FB of *A. bilineatus*.

Sl. No.	Growth Factors and Biologically Active Proteins in FB of *A. bilineatus*	Details of the ELISA Kit Used for the Assay
1	Human Stem Cell Factor (HSCF) [5,33,34]	HSCF ELISA kit, cat. # MBS4502370, MyBioSource, San Diego, CA, USA.
2	Rat Stem Cell Factor (RSCF) [5,33,34]	RSCF ELISA kit, cat. # MBS730168, MyBioSource, San Diego, CA, USA.
3	Human Macrophage Colony Stimulating Factor (HMCSF) [35,36,37]	HMCSF ELISA kit, cat. # MBS165036, MyBioSource, San Diego, CA, USA.
4	Rat Macrophage Colony Stimulating Factor 1 (RMCSF1) [35,36,37]	RMCSF ELISA kit, cat. # MBS008298, MyBioSource Inc., San Diego, CA, USA.
5	Human Vascular Endothelial Growth Factor (HVEGF) [38]	HVEGF ELISA kit, Fine Test, cat. # EH0327, Wuhan Fine Biotech, Wuhan China.
6	Rat Vascular Endothelial Growth Factor (RVEGF) [38]	RVEGF ELISA kit, cat. # EK0540, Boster Biological Technology, Pleasanton, CA 94566, USA.
7	Human Fibroblast Growth Factor 1 (HFGF-1) [39,40]	HFGF1 ELISA kit, catalog # MBS167170, MyBiosource, Inc., San Diego, CA, USA.
8	Rat Fibroblast Growth Factor 1 (RFGF-1) [39,40]	RFGF1 ELISA kit, catalog # MBS705077, MyBiosource, Inc., San Diego, CA, USA
9	Human Fibroblast Growth Factor 19 (HFGF-19) [40,41]	HFGF19 ELISA kit, catalog # MBS938699, MyBiosource, Inc., San Diego, CA, USA.
10	Rat Fibroblast Growth Factor 19 (RFGF-19) [40,41]	RFGF19 ELISA kit, catalog # MBS102206, MyBiosource, Inc., San Diego, CA, USA.
11	Human Fibroblast Growth Factor 21 (HFGF-21) [41,42,43]	HFGF21 ELISA kit, catalog # MBS2023437, MyBiosource, Inc., San Diego, CA, USA
12	Rat Fibroblast Growth Factor 21 (RFGF-21) [41,42,43]	RFGF21 ELISA kit, catalog # MBS2024083, MyBiosource, Inc., San Diego, CA, USA.
13	Human Neutrophil Activating Protein 2 (HNAP-2) [44,45]	HNAP-2 ELISA kit cat. # CSB-E04562h, Wuhan Huamei Biotech Co., Ltd., Wuhan, Hubei Province, China.
14	Rat Neutrophil Activating Protein 2 (RNAP-2) [44,45]	RNAP-2 ELISA kit, cat. # MBS760356, MyBioSource, Inc., San Diego, CA, USA.
15	Human Substance P (HSP) [46,47,48]	HSP ELISA kit, MyBiosource, Cat. # MBS 705162, San Diego, CA, USA.
16	Rat Substance P (RSP) [46,47,48]	RSP ELISA kit, MyBioSource cat. # MBS 703659, San Diego, CA, USA.
17	Human Insulin (HI) [49,50]	HI ELISA kit, My BioSource, cat. # MBS 761338, San Diego, CA, USA.
18	Rat Insulin (RI) [49,50]	RI ELISA kit, My BioSource, cat. # MBS 724709, San Diego, CA, USA.
19	Human Glucagon-like peptide 1 (HGLP-1) [51,52]	HGLP-1 ELISA kit, cat. # EH1053, Wuhan Fine Biotech Co., Ltd., East Lake High Tech Devt. Zone, Wuhan, China.
20	Rat Glucogon-like peptide 1 (RGLP-1) [51,52]	RGLP-1 ELISA kit, cat. # E08117r, Cusabio, Houston, USA.
21	Human Nerve Growth factor (HNGF) [53,54]	HNGF ELISA kit, cat. # MBS2021644, MyBioSource, Inc. San Diego, CA, USA.
22	Rat Nerve Growth Factor (RNGF) [53,54]	RNGF ELISA kit, cat. # MBS775043, MyBioSource, Inc. San Diego, CA, USA.
23	Human Interleukin 19 (HIL-19) [55]	HIL-19 ELISA kit, cat. # MBS 2019896, MyBioSource, San Diego, CA, USA.
24	Rat Interleukin 19 (RIL-19) [55]	RIL-19 ELISA kit, Fine test code: ER1589, Wuhan Fine Biotech Co. Ltd., Wuhan, Hubei, China.
25	Human Interleukin 37 (HIL-37) [56]	HIL-37 ELISA kit, cat. # MBS761932, MyBioSource, San Diego, CA, USA.
26	Rat Interleukin 37 (RIL-37) [56]	Rat IL-37 ELISA kit, cat. # EK720300, AFG Bioscience, 1818 Skokie Boulevard, Northbrook, IL 60062, USA.
27	Human Neurogenin 3 (HNGN-3) [57,58,59]	HNGN-3 ELISA kit, cat. # MBS 2140241, MyBioSource, San Diego, CA, USA.
28	Rat Neurogenin 3 (RNGN-3) [57,58,59]	RNGN-3 ELISA kit, cat. # MBS 2612446, MyBioSource, San Diego, CA, USA.
29	Human Transforming Growth Factor Beta 1 (HTGFβ1) [60]	HTGFβ1 ELISA kit, cat. # ORB 437486, Biorbyt LLC**,** 68 TW Alexander Drive, North Carolina, Durham, 27709, USA.
30	Rat Transforming Growth Factor Beta 1 (RTGFβ1) [60]	RTGFβ1, ELISA Kit, cat. # MBS 824788, MyBioSource, San Diego, CA, USA.
31	Human Hepatocyte Growth Factor (HHGF) [61]	HHGF ELISA kit, cat. # CSB-E04573h, Cusabio Wuhan Huamei Biotech Co., Ltd., Wuhan, Hubei Province 430206, P. R. China.
32	Rat Hepatocyte Growth Factor (RHGF) [61]	RHGF ELISA kit, cat. # CSB-E07346r, Cusabio, Wuhan Huamei Biotech Co., Ltd., Wuhan, Hubei Province 430206, P. R. China.
33	Human Crystallin Beta A1 (HCRYβA1) [62,63]	HCRYβA1 ELISA kit, cat. # MBS 7252187, MyBioSource, San Diego, CA, USA.
34	Mouse Crystallin Beta A1 (MCRYβA1) [62,63]	MCRYβA1 ELISA kit, cat. # MBS 2705771, MyBioSource, San Diego, CA, USA.
35	Human Bone Morphogenetic Protein 3 (HBMP-3) [64]	HBMP-3 ELISA Kit, cat. # MBS 943749, MyBioSource, San Diego, CA, USA.
36	Rat Bone Morphogenetic Protein 3 (RBMP-3) [64]	RBMP-3 ELISA kit, Fine Test, cat. # ER0769 Wuhan Fine Biotech Co., Ltd., Wuhan, 430074, Hubei, China.
37	Human Somatostatin (HSS) [65]	HSS ELISA kit, cat. # BSKH61286, Bioss Antibodies, Bioss Inc., Woburn, MA 01801 USA.
38	Rat Somatostatin (RSS) [65]	RSS ELISA kit, cat. # CSB-E08204r, Cusabio Innovation Centre, Houston, TX 77054, USA.
39	Human Interleukin 15 (HIL-15) [66]	HIL-15 ELISA kit, cat. # MBS 705189, MyBioSource, San Diego, CA, USA.
40	Rat Interleukin 15 (RIL-15) [66]	RIL-15 ELISA kit, cat. # MBS 701942, MyBioSource, San Diego, CA, USA.
41	Human Regenerating Islet Derived Protein 3 Alpha (HREG-3α) [67]	HREG-3α ELISA Kit, cat. # MBS 937542, MyBioSource, San Diego, CA, USA.
42	Rat Regenerating Islet Derived Protein 3 Alpha (RREG-3α) [67]	RREG-3α ELISA kit, cat. # RTEB0672, AssayGenie, 25 Windsor Place, Dublin 2, Ireland, D02 VY42.
43	Human Gamma Amino Butyric Acid (HGABA) [68]	HGABA ELISA kit, cat. # EH3098, Fine Test, Wuhan Fine Biotech Co., Ltd., Wuhan, 430074, Hubei, China.
44	Rat Gamma Amino Butyric Acid (RGABA) [68]	RGABA ELISA kit, cat. # ER1707, Fine Test, Wuhan Fine Biotech Co., Ltd., Wuhan, 430074, Hubei, China.
45	Human Interferon Alpha (HIFN-α) [69]	HIFN-α ELISA Kit, cat. # MBS 702357, MyBioSource, San Diego, CA, USA.
46	Rat Interferon Alpha (RIFN-α) [69]	RIFN-α ELISA Kit, cat. # MBS 2022005, MyBioSource, San Diego, CA, USA.
47	Human Interferon Beta (HIFN-β) [70]	HIFN-β ELISA Kit, cat. # MBS-455331, MyBioSource, San Diego, CA, USA.
48	Rat Interferon Beta (RIFN-β) [70]	RIFN-β ELISA Kit, cat. # MBS 4500062, MyBioSource, San Diego, CA, USA.
49	Human Stem Cell Factor/Mast Cell Growth Factor (HSCF/MGF) [71]	HSCF/MGF ELISA Kit, cat. # MBS 161108, MyBioSource, San Diego, CA, USA.
50	Rat Stem Cell Factor/Mast Cell Growth Factor (RSCF/MGF) [71]	RSCF/MGF ELISA Kit, cat. # MBS 9315966, MyBioSource, San Diego, CA, USA.
51	Human Insulin-like Growth Factor 1 (HIGF-1) [72]	HIGF-1 ELISA Kit, cat. # MBS 014429, MyBioSource, San Diego, CA, USA.
52	Rat Insulin-like Growth Factor 1 Receptor (RIGF-1R) [72]	RIGF-1R ELISA kit, cat. # MBS 062253, MyBioSource, San Diego, CA, USA.
53	Human Prolactin (HPRL) [73]	HPRL ELISA kit, cat. # EH0259, Fine Test, Wuhan Fine Biotech Co., Ltd., Wuhan, 430074, Hubei, China.
54	Rat Prolactin (RPRL) [73]	RPRL ELISA kit, cat. # ER0076, Fine Test, Wuhan Fine Biotech Co., Ltd., Wuhan, 430074, Hubei, China.
55	Human Sirtuin 1 (HSIRT1) [74,75]	HSIRT 1 ELISA kit, cat. # MBS 2601311, MyBioSource, San Diego, CA, USA.
56	Rat Sirtuin 1 (RSIRT1) [74,75]	RSIRT 1 ELISA kit, cat. # MBS 4502504, MyBioSource, San Diego, CA.
57	Human Sirtuin 2 (HSIRT2) [76]	HSIRT2 ELISA kit, cat. # MBS161729, MyBioSource, San Diego, CA, USA.
58	Rat Sirtuin 2 (RSIRT2) [76]	RSIRT2 ELISA kit, cat. # MBS079814, MyBioSource, San Diego, CA, USA.
59	Human Sirtuin 3 (HSIRT3) [77]	HSIRT3 ELISA kit, cat. # MBS 163385, MyBioSource, San Diego, CA, USA.
60	Rat Sirtuin 3 (RSIRT3) [77]	RSIRT 3 ELISA kit, cat. # MBS 047755, MyBioSource, San Diego, CA, USA.
61	Human Sirtuin 4 (HSIRT4) [78]	HSIRT4 ELISA kit, cat. # MBS 452110, MyBioSource, San Diego, CA, USA.
62	Rat Sirtuin 4 (RSIRT4) [78]	RSIRT4 ELISA kit, cat. # MBS 8807375, MyBioSource, San Diego, CA.
63	Human Sirtuin 5 (HSIRT5) [79]	HSIRT5 ELISA kit, cat. # MBS 2705671, MyBioSource, San Diego, CA, USA.
64	Rat Sirtuin 5 (RSIRT5) [79]	RSIRT5 ELISA Kit, cat. # MBS 8807376, MyBioSource, San Diego, CA, USA.
65	Human Sirtuin 6 (HSIRT6) [80]	HSIRT6 ELISA kit, cat. # MBS 162109, MyBioSource, San Diego, CA, USA.
66	Rat Sirtuin 6 (RSIRT6) [80]	RSIRT6 ELISA kit, cat. # MBS 063651, MyBioSource San Diego, CA, USA.
67	Human Sirtuin 7 (HSIRT7) [81]	HSIRT7 ELISA kit, cat. # MBS 2021722, MyBioSource, San Diego, CA, USA.
68	Rat Sirtuin 7 (RSIRT7) [81]	RSIRT7 ELISA kit, cat. # MBS 2705675, MyBioSource, San Diego, CA, USA.
69	Human Growth Hormone (HGH) [82]	HGH ELISA kit, cat. # EH0152, Fine Test, Wuhan Fine Biotech Co., Ltd., Wuhan, 430074, Hubei, China.
70	Rat Growth Hormone (RGH) [82]	RGH ELISA kit cat. # SEA044Ra, Cloud-Clone, 23603 W. Fernhurst Dr., Unit 2201, Katy, TX 77494, USA.
71	Human Interleukin 11 [83]	HIL-11 ELISA kit cat. # MBS2021858, MyBioSource, San Diego, CA, USA.
72	Rat Interleukin 11 [83]	RIL-11 ELISA kit cat. # MBS2887148, MyBioSource, San Diego, CA, USA.
73	Rat Glucagon (RGCG) [84]	RGCG ELISA kit, cat. # MBS2887148, MyBioSource, San Diego, CA, USA.
74	Rat C-Peptide (RC-P) [85]	RC-P ELISA kit, cat. # MBS731074, MyBioSource, San Diego, CA, USA.
75	Human Epidermal Growth Factor (HEGF) [86]	HEGF ELISA kit, cat. # CSB-E08027h, Cusabio Technology LLC, Houston, Texas, USA.
76	Rat Epidermal Growth Factor (REGF) [86]	REGF ELISA kit, cat. # CSB-E08029r, Cusabio Technology LLC, Houston, TX, USA.
77	Human Erythropoietin (HEPO) [87]	HEPO ELISA kit, cat. # MBS2019738, MyBioSource, San Diego, CA, USA.
78	Rat Erythropoietin (REPO) [87]	REPO ELISA kit cat. # CSB-E07323r, CUSABIO, (CUBIO Innovation Center), Houston, TX 77054, USA.
79	Human Platelet-Derived Growth Factor (HPDGF) [88]	HPDGF ELISA kit cat. # MBS 021279, MyBioSource, San Diego, CA, USA.
80	Rat Platelet-Derived Growth Factor (RPDGF) [88]	RPDGF ELISA kit cat. # MBS008389, MyBioSource, San Diego, CA, USA.
81	Rat Connective Tissue Growth Factor (RCTGF) [89]	RCTGF ELISA kit cat. # CSB-E07876r, CUSABIO, CUBIO Innovation Center, Houston, TX 77054, USA.

## Data Availability

All data are available in the manuscript and the Supplementary Materials. Any additional information can be obtained from the corresponding authors.

## Supplementary Materials

The following supporting information can be downloaded at: https://www.mdpi.com/article/10.3390/biom15070929/s1, Figure S1: Graphs show the effects of 12 weeks of FB treatment on blood glucose level, fructosamine level, body weight, and water and food intake in the female diabetic animals. Figure S2: Bar graphs show the effects of 12 weeks of FB treatment on serum insulin, pancreatic insulin and, C-peptide levels in serum of the treated diabetic female animals. Figure S3: Bar graphs show that 12 weeks of FB treatment improved kidney and liver antioxidant profiles of the treated diabetic female animals. Figure S4: Bar graphs show that FB treatment improved the lipid profiles of the treated diabetic female animals.
