# Supplementary material for: Regeneration of Insulin-Producing β Cells, Reduction in Inflammation and Oxidation Stress, and Improvement in Lipid Profile in a Type 1 Diabetes Rat Model by Intraperitoneal Injection of the Growth Factors-Rich Catfish Skin-Derived Fraction-B: An Introductory Report
Source: Biomolecules. 2025 Jun 25;15(7):929. doi: 10.3390/biom15070929 (PMC12292217; doi:10.3390/biom15070929)
Supplement: Supplementary file 1 [file biomolecules-15-00929-s001.zip › biomolecules-3573135-supplementary.pdf]

Supplementary Materials

Manuscript Title:

Regeneration of insulin-producing β-cells, reduction of inflammation, oxidation stress and improvement of lipid profiles in a type 1 diabetes rat model by intraperitoneal injection of the growth factors-rich catfish skin-derived Fraction-B, an introductory report

Table of contents

1. **Introduction**
2. **Materials and Methods**
   2.1- Female experimental animals.
   2.2- Establishment of diabetes in female SD rats and their treatment with FB.
3. **Results**
   3.1. The positive effects of 12 weeks of FB treatment on diabetic female SD rats.
      3.1.1. Effects on blood glucose.
      3.1.2. Effect on fructosamine.
      3.1.3. Effects on body weight and water and food intake.
      3.1.4. FB treatment improved insulin level in serum and pancreas and C-peptide in serum of female diabetic animals.
      3.1.5. FB-treated diabetic female animals displayed improved oxidation/antioxidation profiles.
      3.1.6. FB treatment improved the serum lipid profiles of diabetic female rats.
4. **Conclusion**

1. Introduction

Male animals are preferred to female animals in biological experiments because of the possibility of interference from female hormones. In this study, we used male SD rats first. We established that injured islet recovery and β-cell regeneration could be achieved after IP injection of FB into male diabetic animals. We tested the effects of FB on diabetic female SD rats to see whether FB has the same effect on the females as it had in the male. Hence, several of the experiments that were performed on diabetic male SD rats were repeated in diabetic female SD rats using procedures similar to those applied to the male animals, as described in the Methods. In this study, female rats were subjected to IP injection of FB for 12 weeks, instead of the 8-week treatment period for male rats. This was because of difficulties that arose with working on the animals during the COVID-19 pandemic. The following experiments were conducted in female SD rats.

2. Methods

2.1.Female experimental animals

In this section of our study, healthy female SD rats three-months of age weighing 150-180 g were used. Animal experiments and experimental procedures were an exact repeat of those conducted in the male group of animals and described in Materials and methods. The animals were kept at a constant temperature (23°C ± 2°C) and humidity on a 12-hour light/dark cycle. Rats were housed individually in separate cages and were provided food and water *ad libitum* throughout the experiment. A typical experiment included 31 rats that were randomly grouped into three groups: 7 rats in normal control (NC), 12 rats in diabetic control (DC), and 12 diabetic rats treated with FB (D+FB) as described below. The animal experiments followed the procedures of the Animal Ethics Committee of Kuwait University Health Sciences Centre, and the study was carried out under US guidelines (NIH Publication #85-23, revised in 1985). All efforts were made to minimize the number of animals used in the study and their suffering.

2.2.Establishment of diabetes in female SD rats and their treatment with FB

Diabetes was induced in female SD rats by IP injection of a single dose of 60 mg STZ/kg body weight in citrate buffer (0·01 M, pH 4·5) within 5 min of its preparation [21,22]. One week after STZ injection, blood glucose (BG) was measured, and rats with BG levels > 15 mmol/L were considered diabetic. Diabetic rats were randomly divided into two groups. The rats in the diabetic control (DC) group were treated daily with PBS (pH 7·5) via IP injection. The volume varied for each animal according to its weight as if it were to be injected with FB in PBS. The rats in the D+FB group were IP injected daily with FB [0·3 mg/100 g body weight] in PBS (pH 7·5) daily for 12 weeks. Rats in the normal control (NC) group were not diabetic (had not been treated with STZ), each was injected with the appropriate volume of PBS (pH 7·5) according to its weight, as was the case for the DC group. All animal groups were treated at the same time of day.

3. Results

3.1.FB treatment of diabetic female rats led to improvement in diabetes symptoms

3.1.1. Improvement in blood glucose level: The blood glucose level in female DC animals showed a gradual and significant increase ($p < 0.05$) throughout the 12 weeks. However, the D+FB female animals showed a steady and significant ($p < 0.05$) reduction in blood glucose, reaching its lowest level in 12 weeks when the animals were sacrificed (Figure S1 A).

3.1.2. Improvement in fructosamine level: Similarly, compared with untreated diabetic animals, FB-treated diabetic female animals exhibited a significant decrease in fructosamine levels (Figure S1 B).

3.1.3. Improvement in body weight and water and food intake: Additionally, compared with diabetic animals, FB-treated female diabetic animals showed significant improvements in body weight (Fig. S1 C) and concomitant reductions in water and food intake (Figures S1 D and S1 E respectively). All the results were significantly different ($p < 0{\cdot}05$) from those of the DC animals.

A

B

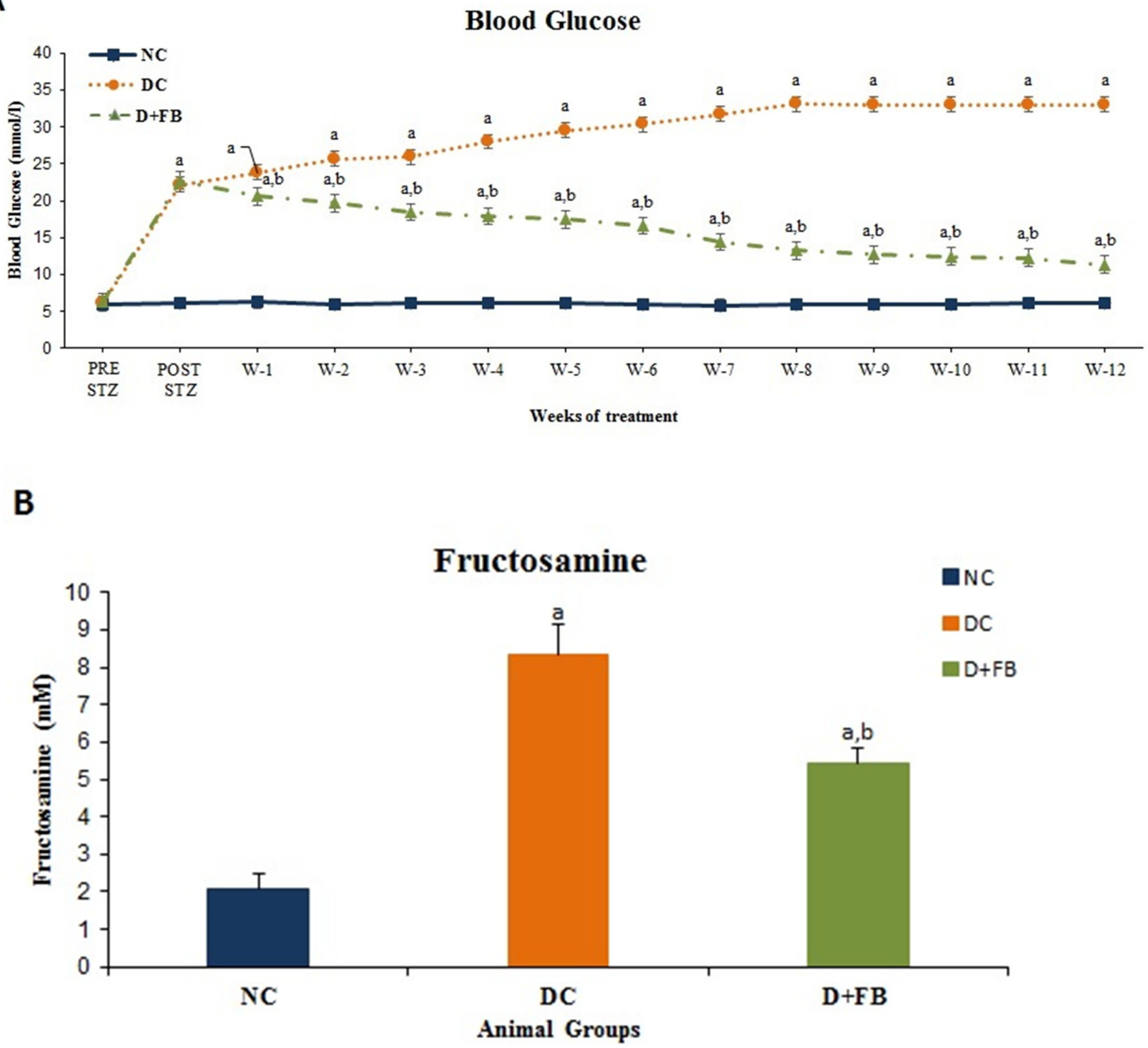

Blood Glucose
NC
DC
D+FB
Blood Glucose (mmol/l)
Weeks of treatment
PRE STZ
POST STZ
W-1
W-2
W-3
W-4
W-5
W-6
W-7
W-8
W-9
W-10
W-11
W-12
Fructosamine
Fructosamine (mM)
Animal Groups

C

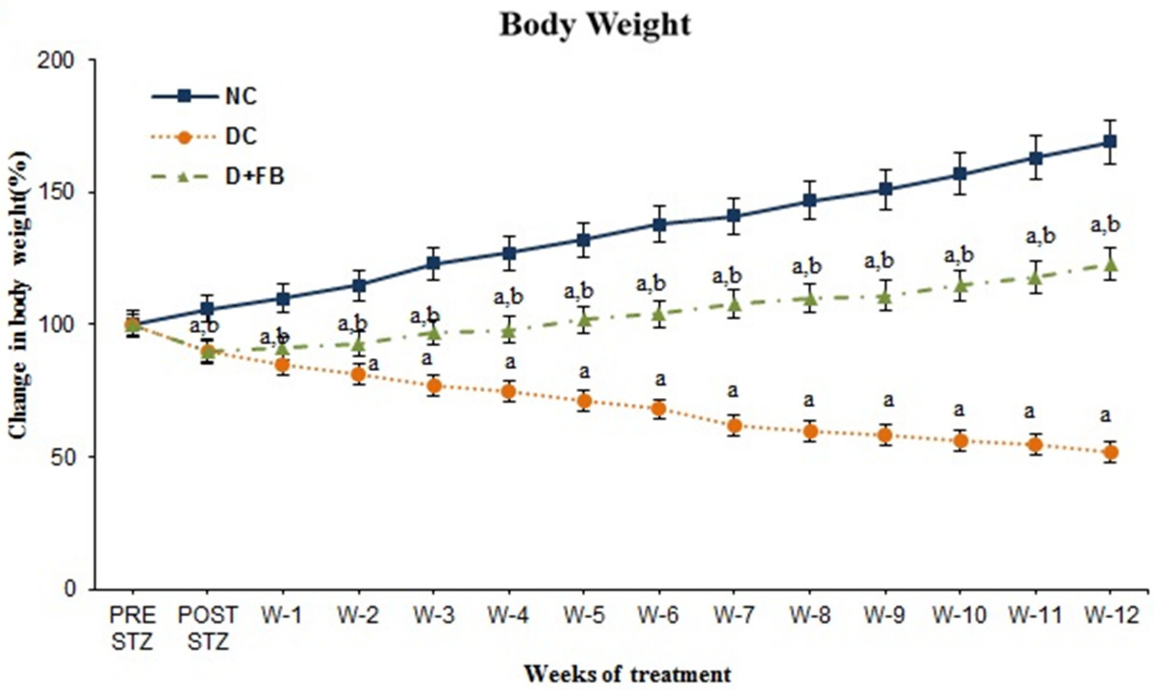

Body Weight
NC
DC
D+FB
Change in body weight(%)
200
150
100
50
0
a,b
a
PRE STZ
POST STZ
W-1
W-2
W-3
W-4
W-5
W-6
W-7
W-8
W-9
W-10
W-11
W-12
Weeks of treatment

D

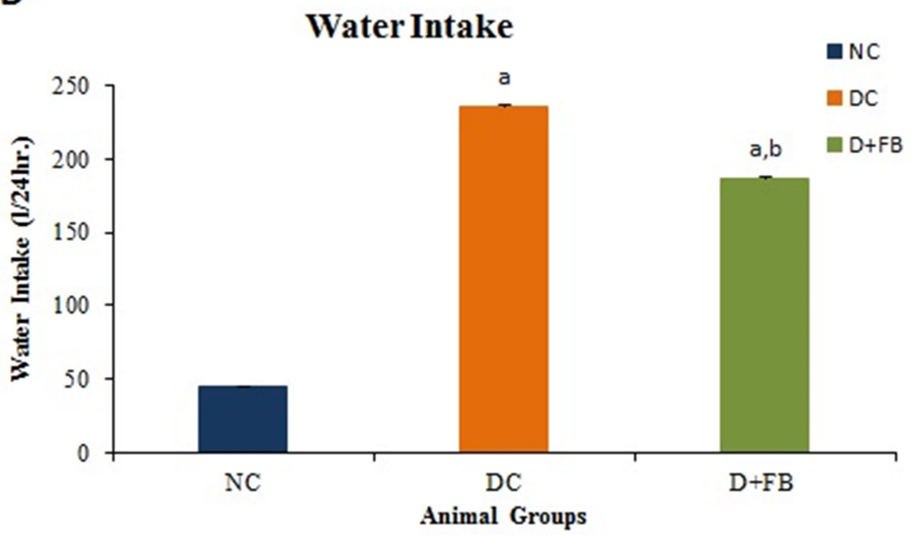

Water Intake
NC
DC
D+FB
Water Intake (l/24hr.)
250
200
150
100
50
0
a
a,b
NC
DC
D+FB
Animal Groups

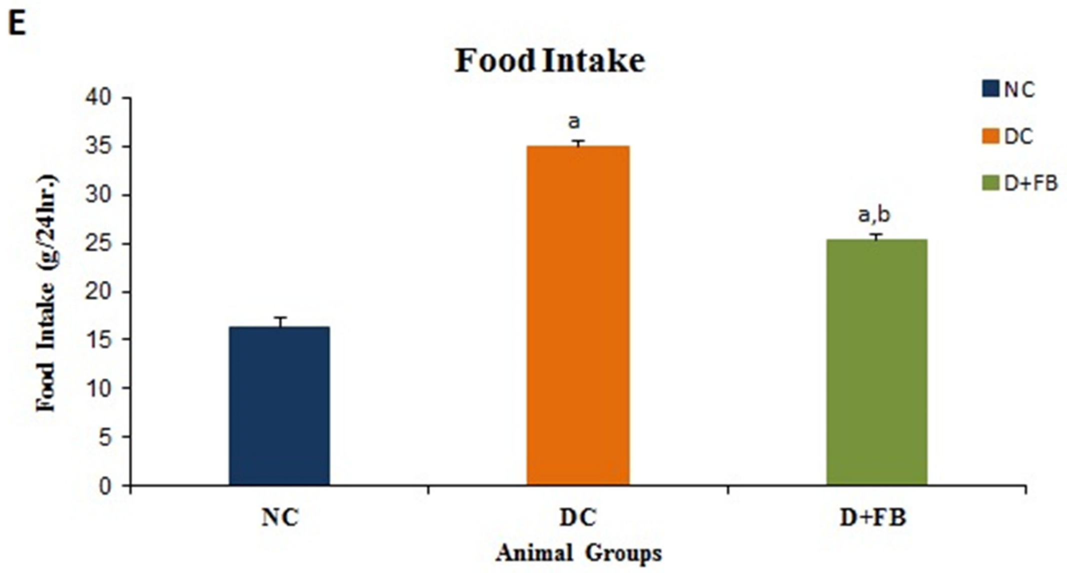

Figure S1. A –E:

Graphs show the effects of 12 weeks of FB treatment on blood glucose level, fructosamine level, body weight, and water and food intake in the female diabetic animals.

(A): A line graph shows reduction in blood glucose level; **(B)** A bar graph shows reduction in fructosamine level; **(C)** A line graph shows improvement in body weight; **(D)** a bar graph shows reduction in water intake and **(E)** A bar graph shows the reduction in food intake in the D+FB-treated female rats. All the graphs are significant ($P < 0{\cdot}05$) compared to those in the DC and NC rats at the end of 12 weeks of FB treatment.

These results show that FB acts equally on both diabetic male and female rats to provide similar functional recovery of the diabetic pancreas.

3.1.4. FB treatment improved insulin levels in serum and pancreatic tissue and C-peptide in serum of the diabetic female animals:

The DC female animals showed a 94% reduction in the serum insulin concentration compared to that of the NC group, while the FB-treated female animals showed a significant increase (59%) in the insulin concentration compared to that of the diabetic control female rats (Figure S2 A). Similarly, the pancreatic tissue insulin concentration of the diabetic female rats was significantly lower (66%) than that of the NC group, while the pancreatic tissue insulin concentration was significantly greater (50%) in the FB-treated female animals than in the DC female animals (Figure S2 B). The concentration of C-peptide in the serum of the FB-treated diabetic rats was significantly higher than in that of the DC diabetic rats, which confirmed that insulin was produced by the newly regenerated β cells after FB treatment (Figure S2 C). All the results were significantly different ($p < 0{\cdot}05$) from those of the DC animals.

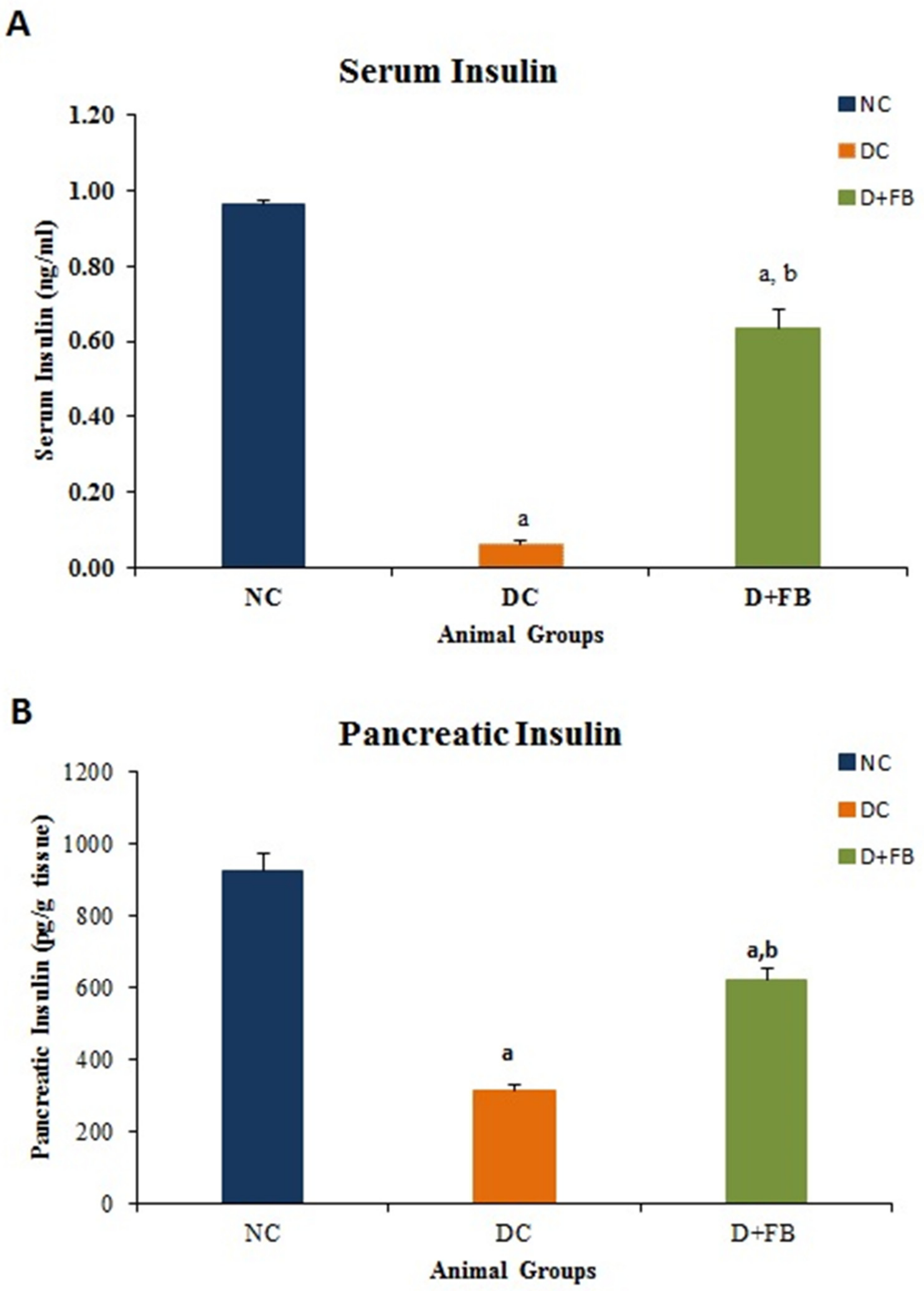
A
Serum Insulin
Serum Insulin (ng/ml)
1.20
1.00
0.80
0.60
0.40
0.20
0.00
a
a, b
NC
DC
D+FB
Animal Groups
NC
DC
D+FB
B
Pancreatic Insulin
Pancreatic Insulin (pg/g tissue)
1200
1000
800
600
400
200
0
a
a,b
NC
DC
D+FB
Animal Groups
NC
DC
D+FB

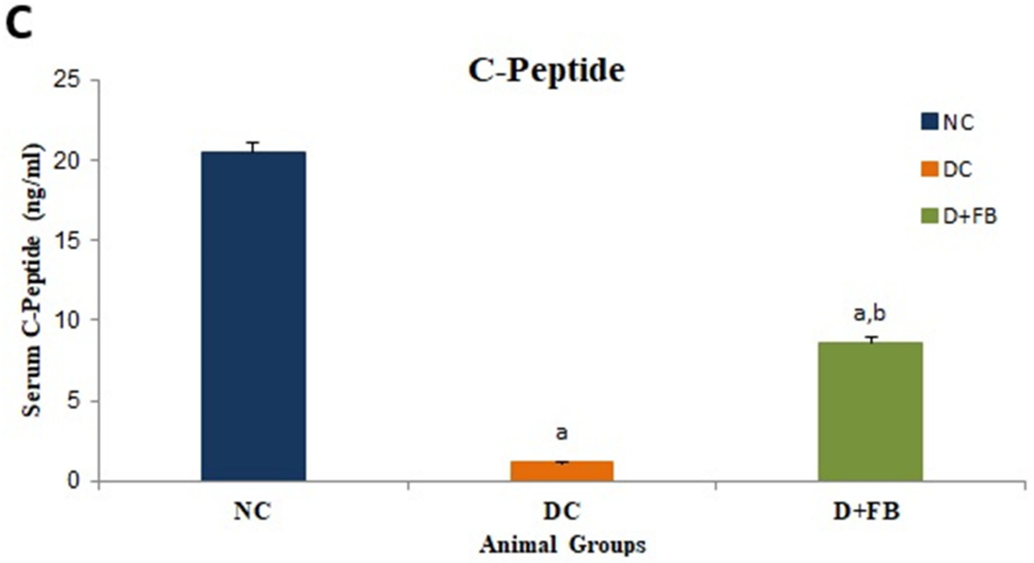

Figure S2. A-C: Bar graphs show the effects of 12 weeks of FB treatment on serum insulin, pancreatic insulin and, C-peptide levels in serum of the treated diabetic female animals.

(A): A bar graph shows concentration of insulin in the serum; **(B)** A bar graph shows concentration of insulin in the pancreases and **(C)** A bar graph shows the concentration of C-Peptide in the serum of the D+FB-treated female rats compared to those of the NC and DC animals. All the results are significant ($P < 0{\cdot}05$) compared to those in the DC and NC rats.

Compared with NC animals, female DC animals showed a significant ($p < 0{\cdot}05$) reduction in insulin levels in the serum and pancreatic tissue. However, FB treatment significantly ($p < 0{\cdot}05$) increased the insulin levels in the serum and in pancreatic tissue in the 12 weeks FB-treated female animals compared to those in the serum of diabetic female DC animals. FB treatment significantly ($p < 0{\cdot}05$) increased the serum C-peptide concentration in diabetic rats, confirming that insulin was produced by the newly regenerated β-cells.

These results showed that FB had an equal effect on both diabetic male and female rats and promoted recovery of the diabetic pancreas.

3.1.5. FB-treated diabetic female animals displayed improved oxidation/antioxidation profiles:

The antioxidant components of FB, such as F-acids, cholesta-3,5-diene, IL-37, IL-11 and IL-19 were expected to improve the oxidation/antioxidation profile of FB-treated female rats. Analysis of the oxidation/antioxidation profiles of the homogenized female kidneys following 12 weeks of treatment revealed similar results to those obtained for the FB-treated male animals. Briefly, the FB-treated animals showed a significant reduction in the kidney MDA level compared to that in the DC group, while the antioxidant and catalase levels significantly increased in the kidneys of the FB-treated diabetic animals compared to those in the DC group (Figure S3 A). All the results were significantly different ($p < 0{\cdot}05$) from those of the DC animals and were similar to those for the diabetic male animals (Figure 5 A).

Likewise, analysis of the oxidation/antioxidation profile in the livers from the female groups following 12 weeks of treatment revealed a similar recovery to that shown for the male animals (Figure 5 B). Briefly, the FB-treated animals showed a significant reduction in the liver MDA level compared to that in the DC group, while the antioxidant and catalase levels significantly increased in the livers of FB-treated diabetic animals compared to those in the DC group (Figure S3 B). All the results were significantly different ($p < 0{\cdot}05$) from those of the DC animals.

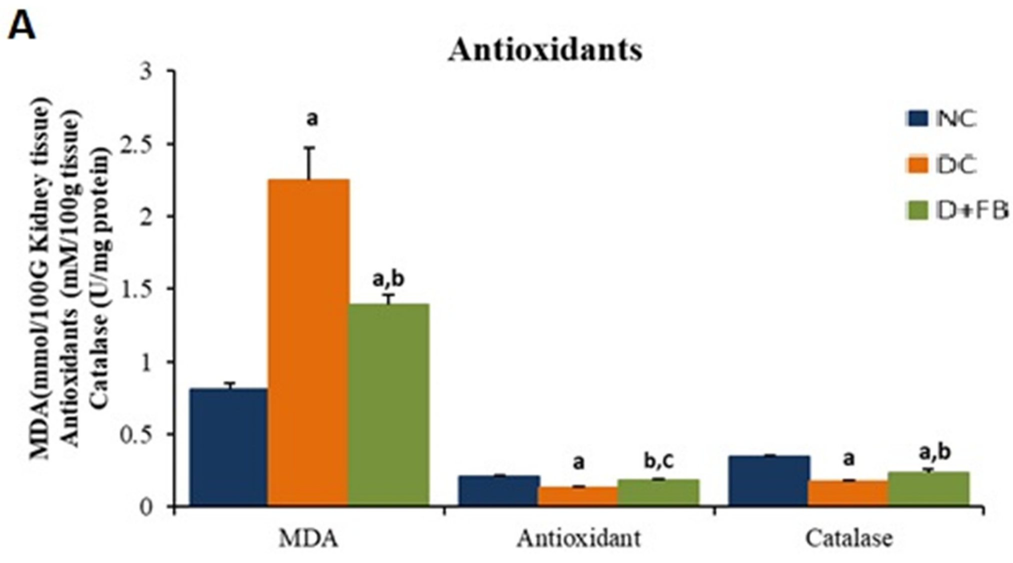

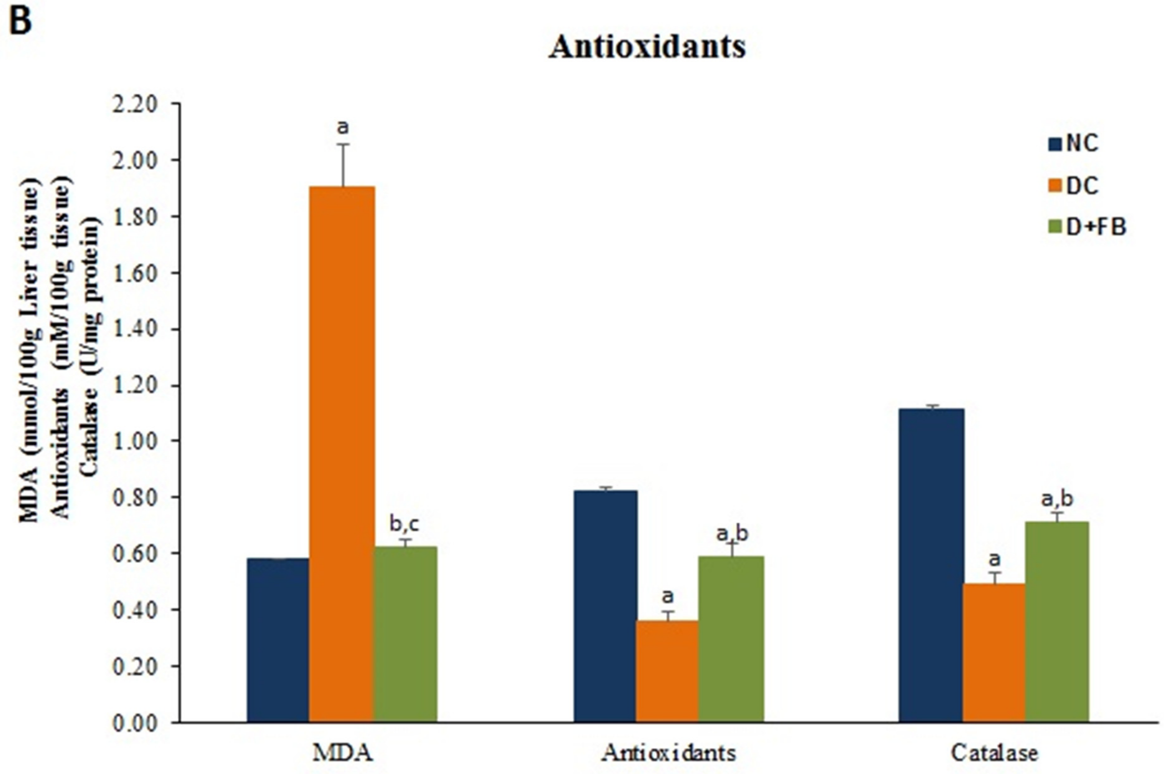

Figure S3. A–B: **Bar graphs show that 12 weeks of FB treatment improved kidney and liver antioxidant profiles of the treated diabetic female animals.**
(A): A bar graph shows the benefit of FB treatment on kidney antioxidant parameters; **(B)** A bar graph shows the benefit of FB treatment on liver antioxidant parameters in the serum of the D+FB-treated female rats. All the graphs are significant ($P < 0{\cdot}05$) compared to those in the DC and NC rats at the end of 12 weeks of FB treatment.

The oxidation/antioxidation profiles of the kidney and liver of the three experimental female groups following 12 weeks of FB treatment shows that the FB-treated animals showed a significant ($p < 0{\cdot}05$) reduction in the MDA level of kidney and liver compared to that in the DC animals, while the antioxidant and catalase levels of kidney and liver significantly ($p < 0{\cdot}05$) increased in the FB-treated female animals compared to those in the DC animals thus improving oxidative stress.

3.1.6. FB treatment improved the serum lipid profiles of diabetic female animals.

The blood sera of the diabetic female SD rats treated for 12 weeks with FB were analysed to determine lipid (cholesterol, HDL, LDL, and triglyceride) concentrations. The lipid analysis results were compared with those obtained for N and DC animals. The FB-treated female rats showed improvements in their serum lipid profile (Figure S4). All the results were significantly different ($p < 0{\cdot}05$) from those of the DC animals. These results show that FB acts equally on both diabetic male (Figure 7) and diabetic female rats to improve the lipid profile.

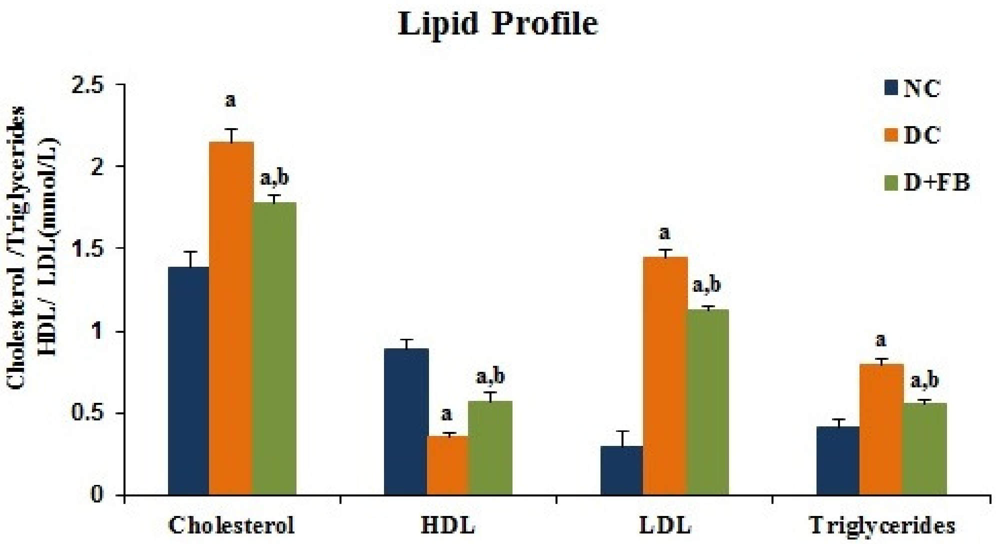

Figure S4: Bar graphs show that FB treatment improved the lipid profiles of the treated diabetic female animals.
Bar graphs show the concentrations of serum lipids in the three groups (NC, DC and D+FB) of female rats. The bar graphs show the significant ($p < 0{\cdot}05$) improvements in the serum lipid profile (cholesterol, HDL, LDL, and triglycerides) of the FB-treated (D+FB) diabetic female rats compared to that of the DC animals.

4. Conclusion:

Experiments conducted on diabetic female animals resulted in the recovery of the diabetic pancreas and health quality improvement. These results are similar to those obtained for the action of FB on the diabetic male animals, hence FB acts equally on both diabetic male and female animals.
